# A self-generated Toddler gradient guides mesodermal cell migration

**DOI:** 10.1126/sciadv.add2488

**Published:** 2022-09-14

**Authors:** Jessica Stock, Tomas Kazmar, Friederike Schlumm, Edouard Hannezo, Andrea Pauli

**Affiliations:** ^1^ Research Institute of Molecular Pathology (IMP), Vienna Biocenter (VBC), Vienna, Austria.; ^2^ Vienna BioCenter PhD Program, Doctoral School of the University of Vienna and Medical University of Vienna, Vienna, Austria.; ^3^ Institute of Science and Technology Austria (IST), Klosterneuburg, Austria.

## Abstract

The sculpting of germ layers during gastrulation relies on the coordinated migration of progenitor cells, yet the cues controlling these long-range directed movements remain largely unknown. While directional migration often relies on a chemokine gradient generated from a localized source, we find that zebrafish ventrolateral mesoderm is guided by a self-generated gradient of the initially uniformly expressed and secreted protein Toddler/ELABELA/Apela. We show that the Apelin receptor, which is specifically expressed in mesodermal cells, has a dual role during gastrulation, acting as a scavenger receptor to generate a Toddler gradient, and as a chemokine receptor to sense this guidance cue. Thus, we uncover a single receptor–based self-generated gradient as the enigmatic guidance cue that can robustly steer the directional migration of mesoderm through the complex and continuously changing environment of the gastrulating embryo.

## Teaser

Aplnr has a dual role to self-generate and sense a Toddler gradient directing mesodermal cells during zebrafish gastrulation.

## INTRODUCTION

An animal’s body plan is first laid out during gastrulation, which assembles the three germ layers—mesoderm, endoderm, and ectoderm—through the combination of cell migration and differentiation (*1*–*3*). While research throughout the past few decades has provided fundamental insights into how the germ layers are specified (*3*–*6*), our knowledge of the molecular mechanisms that direct their spatial organization is very limited.

In zebrafish embryos, gastrulation starts with Nodal-induced specification and internalization of mesendodermal progenitor cells at the blastoderm margin (*1*, *7*, *8*). Internalized cells subsequently migrate toward the animal pole, giving rise to the mesodermal and endodermal germ layers (*3*, *9*). The molecular guidance, particularly of ventrolateral mesoderm migration, has remained largely unknown across vertebrates. The only factor currently known to be required for this process is the secreted protein Toddler/ELABELA/Apela (*10*, *11*). Toddler is required primarily for mesoderm migration, acting through the Apelin receptor (in zebrafish, Apelin receptor a and b, collectively referred to as Aplnr), a heterotrimeric guanine nucleotide–binding protein (G protein)–coupled receptor (GPCR) specifically expressed in mesodermal cells (*10*–*12*). However, how Toddler establishes directional migration during gastrulation has remained unclear.

Here, we show that mesodermal cells provide a dynamic sink to self-generate a Toddler gradient that acts as their elusive guidance cue. We find that the Aplnr is the sole mediator of both Toddler gradient formation and sensing, which unveils a previously unknown mode of gradient self-generation in an in vivo system. Altogether, our study uncovers a simple yet robust mechanism that guides mesodermal cells through the dynamic and complex environment of a gastrulating embryo.

## RESULTS

### Toddler acts cell non-autonomously to attract Aplnr-expressing cells

**Fig. 1.**
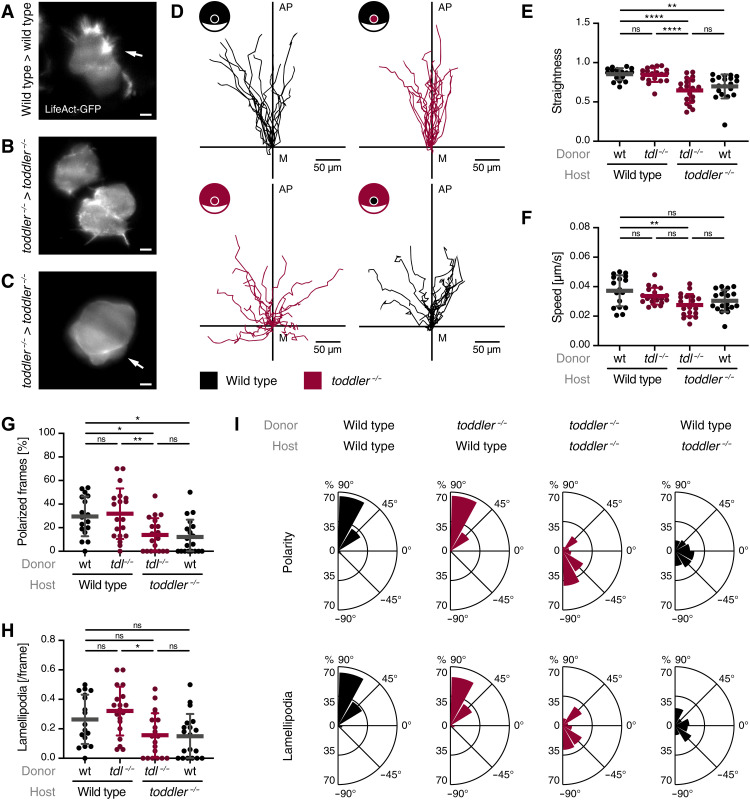
Toddler acts cell non-autonomously to mediate animal pole–directed polarization and migration of mesendodermal progenitors. LifeAct-GFP–labeled reporter cells (maximum 10 cells) were transplanted from the margin of a wild-type or *toddler^−/−^* donor embryo to the margin of a host embryo of the same or opposite genotype. (**A** to **C**) Representative light sheet microscopy images of internalized wild-type (A) or *toddler^−/−^* (B and C) reporter cells in a genotype-matched host embryo. White arrows indicate lamellipodia (A) and blebs (C). Scale bars, 10 μm. (**D**) Migration tracks of transplanted reporter cells [*n* = 19 cells, except for wild type in wild type (*n* = 17)]. Genotypes of donor cells and host embryos are indicated in the embryo scheme (black: wild type; red: *toddler^−/−^*). *x* axis = margin; *y* axis = animal-vegetal axis; coordinate origin = start of track. (**E**) Quantification of track straightness. (**F**) Quantification of migration speed of cells tracked in (D). (**G**) Quantification of cell polarity of internalized cells represented as the percentage of frames in which a cell was polarized. (**H**) Quantification of lamellipodia represented as average number of lamellipodia detected per frame. (**I**) Rose plots showing relative enrichments of orientations of polarity and lamellipodia. Data are means ± SD. Significance was determined using one-way analysis of variance (ANOVA) with multiple comparison; ****P < 0.0001; **P < 0.01; *P < 0.05; n.s., not significant. Rose plots: 90° = animal pole; 0° = ventral/dorsal; −90° = vegetal pole. All graphs are oriented with the animal pole toward the top.

We had previously shown that ventrolateral mesendoderm fails to migrate to the animal pole in *toddler^−/−^* embryos (*10*), yet the underlying cause remained unclear. To unveil the role of Toddler in mesendodermal cell migration, we first assessed the migratory behavior of mesendodermal progenitors. To this end, we transplanted between one and five marginal cells from a LifeAct–green fluorescent protein (GFP)–expressing wild-type or *toddler^−/−^* donor embryo to an unlabeled, stage- and genotype-matched host embryo and tracked cells after internalization using either light sheet (Fig. 1, A to C; fig. S1; and movies S1 to S3) or confocal microscopy (Fig. 1, D to I; fig. S2; and movies S4 and S5). Consistent with previous observations (*2*, *10*, *13*, *14*), wild-type cells migrated in a directional manner from the margin toward the animal pole, were polarized, and extended actin-rich protrusions toward the animal pole (Fig. 1, A and D to I; fig. S1; and movies S1 and S4). In contrast, internalized *toddler^−/−^* cells in *toddler^−/−^* embryos displayed nondirectional migration and were often dragged along with epiboly movements because of reduced and randomly oriented polarity and lamellipodia formation, concomitant with an increased frequency of cell blebbing (Fig. 1, B to I; figs. S1 and S2; and movies S2, S3, and S5). Given that higher cell density can increase the frequency of blebbing (*15*–*17*), this transition from actin- to bleb-based protrusions is likely a secondary effect that can be attributed to the increased cell density at the margin in *toddler^−/−^* mutant embryos. Together, these results reveal that, while *toddler^−/−^* cells are able to migrate in the absence of Toddler, they lack directionality toward the animal pole.

Given that Toddler is a secreted protein (*10*, *11*), we hypothesized that it regulates directional migration of mesodermal progenitors via a cell non-autonomous mechanism. To distinguish cell-intrinsic from cell-extrinsic effects, we transplanted marginal cells from wild-type or *toddler^−/−^* donor embryos into unlabeled, stage-matched host embryos of the opposite genotype. *Toddler^−/−^* cells placed into a wild-type host embryo were able to migrate directionally, polarize, and extend actin-rich protrusions toward the animal pole (Fig. 1, D to I, and movie S6). Wild-type cells in a Toddler-deficient environment, on the other hand, displayed the *toddler^−/−^* phenotype and failed to migrate directionally away from the margin (Fig. 1, D to I; fig. S2; and movie S7). Combined, these results confirm that Toddler acts cell non-autonomously to mediate the migration of mesendodermal progenitors.

**Fig. 2.**
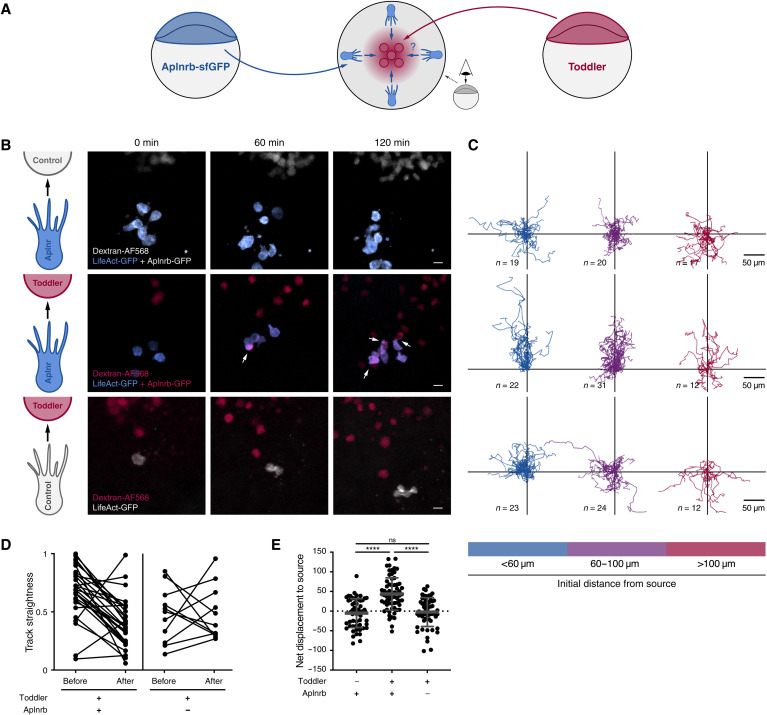
Aplnr-expressing cells are attracted toward a local source of Toddler. (**A**) Schematic representation of the experimental setup to test for Toddler functioning as a chemokine signal for Aplnr-expressing cells. Toddler-expressing cells, red; Aplnrb-expressing cells, blue. (**B**) Snapshots of a time-lapse confocal imaging series assessing the ability of Aplnrb-sfGFP–expressing toddler^−/−^ cells to react to an ectopically located Toddler or control source. Top: Exposure of Aplnr-sfGFP–expressing cells (blue) to Toddler-deficient control cells (gray). *n* = 56 cells. Middle: Exposure of Aplnr-sfGFP–expressing cells to Toddler-overexpressing source cells (red). *n* = 65 cells. White arrows indicate contact between Aplnrb-sfGFP–expressing cells and Toddler source cells. Bottom: Exposure of cells deficient in Aplnr expression (gray) to Toddler-overexpressing source cells (red). *n* = 59 cells. (**C**) Cell tracks corresponding to conditions described in (B). Tracks were grouped by the distance of the cell from the source at the start of imaging (blue: <60 μm, purple: 60 to 100 μm, red: >100 μm). (**D**) Quantified track straightness of all Aplnrb-sfGFP–expressing and Aplnr-deficient cells that reach direct cell-cell contact with a Toddler-expressing source cell. Track straightness was compared before and after contact with the source cell. (**E**) Quantification of net displacement toward the source. All graphs and images are oriented with the source at the top.

A cell non-autonomous signaling mechanism, as well as the loss of directional migration and polarity in the absence of Toddler, indicates that Toddler could act as a chemokine to guide mesodermal cells to the animal pole. As previous efforts to unravel the role of Toddler have led to contradictory results, providing support for both a chemoattractant (*18*) and a motogen (*10*) function, we revisited this question and assessed the ability of Toddler to attract Aplnrb-expressing cells. Aplnrb-sfGFP–expressing cells from a *toddler^−/−^* donor embryo transplanted next to a source of Toddler at the animal pole of a stage-matched *toddler^−/−^* host embryo (Fig. 2, A and B) displayed directional migration toward a Toddler-expressing source until they made initial contact with and attached to Toddler-expressing cells (Fig. 2, B to E; fig. S3; and movie S8). This cellular behavior is similar to the one described for the previously reported chemokine receptor/ligand pair Cxcr4b/Cxcl12a (*19*). Directional migration was induced robustly over a distance of 100 μm (Fig. 2C) and required both the ligand and receptor to be present because the lack of either Toddler in the source or Aplnr in the migrating cells (knockdown using *aplnra/b* morpholinos) led to the loss of directional migration (Fig. 2, B to E, and movie S8) and reduced contacts between Aplnrb-sfGFP–expressing and Toddler-expressing cells (fig. S3B and movie S8). Together, these results indicate that Toddler can provide a chemoattractant signal and that Aplnr is necessary as a chemokine receptor to receive the signal.

### Mesodermal cells are guided by a self-generated Toddler gradient

**Fig. 3.**
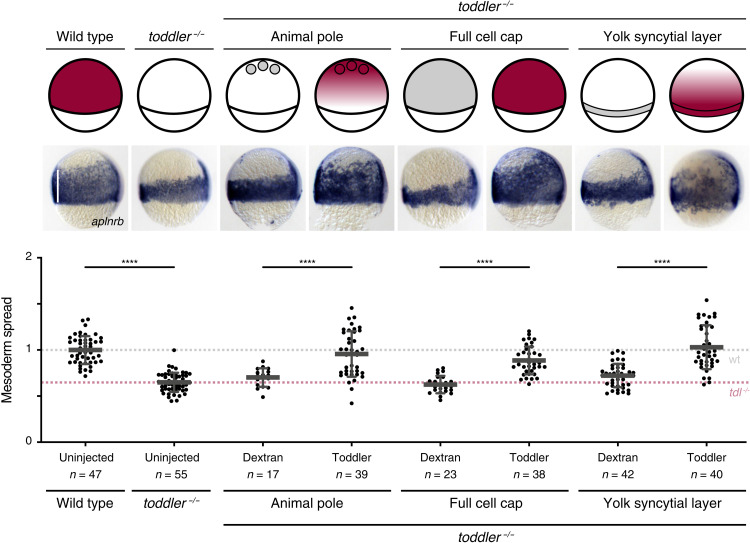
Rescue of the *toddler^−/−^* phenotype is independent of the Toddler sources’ location. Ectopic expression of Toddler in *toddler^−/−^* embryos according to the schematic representation of embryos at 75% epiboly (top). Animal pole source: transplantation of Toddler-overexpressing cells to the animal pole of sphere stage *toddler^−/−^* embryos; ubiquitous expression: injection of 2 pg of *toddler* mRNA (rescuing concentration) into one-cell stage *toddler^−/−^* embryos; marginal source: injection of 10 pg of *toddler* mRNA into the YSL of 1k-cell stage *toddler^−/−^* embryos. Toddler (red); Dextran control (gray). Mesoderm spread was assessed using in situ hybridization for *aplnrb* (middle; lateral view, dorsal on the right; white line indicates measurement of ventral mesoderm spread) and quantified relative to the average spread in wild-type embryos (bottom). Data are means ± SD. Significance was determined using one-way ANOVA with multiple comparison; ****P < 0.0001; **P < 0.01. Scale bars, 20 μm.

Our results so far indicate that Toddler can act as a guidance cue that attracts mesodermal cells. In support of this, placing a cluster of Toddler-expressing cells at the animal pole is sufficient to restore animal pole–directed mesoderm migration in *toddler^−/−^* embryos (Fig. 3). However, in line with previous results (*10*), expression of Toddler throughout the embryo cap, leading to its homogeneous distribution, or from the margin [*toddler* mRNA injection into the yolk syncytial layer (YSL) before the onset of gastrulation], causing Toddler to be graded in the opposite direction, is also able to rescue animal pole–directed mesoderm migration in *toddler^−/−^* embryos (Fig. 3). These results are contrary to typical chemokine models based on concentration gradients formed by a fixed source or sink and suggest that mesoderm migration during gastrulation requires Toddler but is largely independent of Toddler’s expression site.

**Fig. 4.**
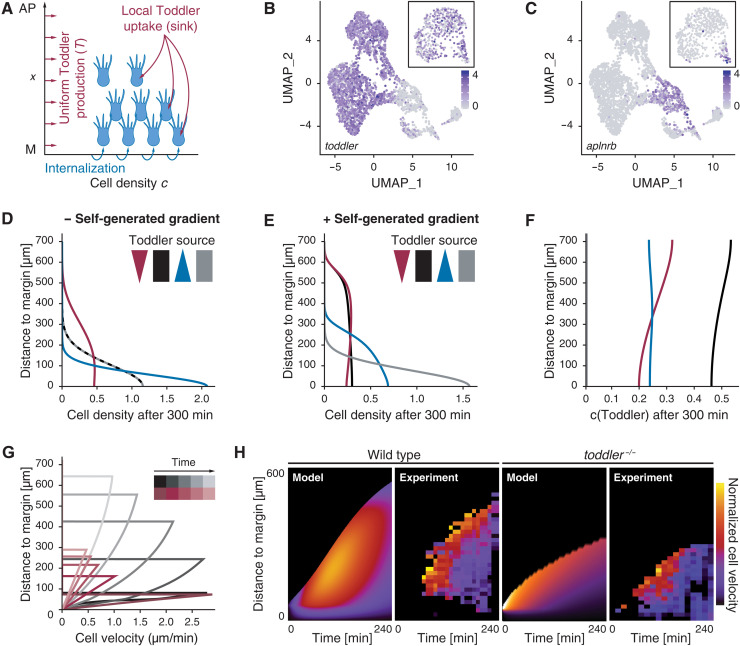
Computational simulations predict a self-generated Toddler gradient. (**A**) Schematic representation of the one-dimensional model of mesoderm density and Toddler concentration along the animal-vegetal axis (*x* = 0 denotes the margin, at which mesodermal cells are added). Toddler (red) is produced uniformly at rate *T*_0_ and is degraded locally by mesodermal cells (blue). (**B** and **C**) Uniform manifold approximation and projection (UMAP) clustering of single cells at 60% epiboly based on scRNA-seq data (*24*). Inset depicts UMAP at 30% epiboly. Color code represents expression levels of *toddler* (B) and *aplnrb* (C) in individual cells. (**D** and **E**) Predicted mesoderm density profiles (arbitrary units) after 300 min without (D) or with (E) Toddler uptake by mesoderm, for different profiles of Toddler production *T*_0_(*x*): graded toward the margin (blue), graded toward the animal pole (red), uniform (black), or no production (gray). (**F**) Predicted Toddler concentrations (arbitrary units) after 300 min with Toddler uptake by mesoderm. Profiles of Toddler productions *T*_0_(*x*) as described in (D) and (E). (**G**) Predicted spatiotemporal profiles of mesodermal cell velocities in wild-type (black) and *toddler^−/−^* embryos (red). (**H**) Predicted (model) and experimental (experiment) kymographs of mesodermal cell migration in wild-type (left) and *toddler^−/−^* (right) embryos. Experimental data from light sheet microscopy and tracking of *drl:GFP*-positive cells [*N* = 7 (wild type) and *N* = 6 (*toddler^−/−^*) embryos; average number of *n* = 195 cells tracked per embryo]. Color code represents normalized velocity (yellow: high; dark purple: low).

Studies in the zebrafish lateral line (*20*, *21*) and *Dictyostelium* (*22*, *23*) have shown that migrating cells can “self-generate” chemokine gradients by locally taking up the chemokine, making them independent of a localized source. To assess quantitatively whether mesodermal cells could self-polarize by locally shaping a Toddler gradient, we turned to computational modeling (see Supplementary Materials). We simulated the one-dimensional migration of mesodermal cells along the animal-vegetal axis *x* with local cell density *c*(*x,t*) over time (Fig. 4A, see also fig. S4, A to E, for a sensitivity analysis and Supplementary Materials for details). Cells internalize at the margin (*x* = 0), can randomly diffuse (random movements with diffusion coefficient *D_c_*), and migrate directionally according to the local gradient of the Toddler concentration profile *T*(*x,t*). Simulation of the Toddler concentration *T*(*x,t*) along the animal-vegetal axis factored in random Toddler diffusion (with coefficient *D_T_*), baseline degradation (with time scale τ*_T_*), and a sink to remove Toddler that is proportional to the local density of mesodermal cells (under the simplifying assumption that each cell has a constant capacity to remove Toddler, see Supplementary Materials for details and extensions of the model to more general situations). We also took into consideration that the quantity of available Toddler is not a fixed value, as in typical in vitro assays of self-generated gradients (*22*, *23*). Based on single-cell RNA sequencing (scRNA-seq) data (*24*), *toddler* mRNA is expressed ubiquitously throughout the embryo at the onset of gastrulation (Fig. 4B) and becomes restricted to nonmesodermal cells during gastrulation [indicated by the mutually exclusive expression patterns of *toddler* and *aplnrb* (Fig. 4, B and C)]. On the basis of these observations, we infer that Toddler is continuously and homogeneously expressed along the animal-margin axis (e.g., by the overlying ectodermal tissue) throughout gastrulation.

To test the core assumptions of the model and constrain its parameters, we first measured the baseline degradation time τ*_T_* of Toddler. To this end, we injected in vitro–synthesized Toddler peptide into *toddler^−/−^, MZoep^−/−^* double-mutant embryos deficient for both Toddler production (*toddler^−/−^*) and the proposed mesodermal Toddler sink (*MZoep^−/−^*) (*25*, *26*). We found that a single time scale can describe Toddler degradation kinetics in *MZoep^−/−^, toddler^−/−^* double mutants, from which we can extract the parameter τ*_T_* ≈ 2h (fig. S4, F and G). Given the known molecular weight of Toddler, we could also estimate its random diffusion to be *D_T_* ≈ 3.10^3^μm^2^/min [a typical value for small diffusible molecules, see (*27*) and Supplementary Materials]. On the basis of these calculations, locally produced Toddler can propagate up to length scales of $\sqrt{D_{T}\tau_{T}}\approx 600\;\mu m$ (the typical size of a zebrafish embryo) in the absence of a mesoderm sink. Together, these measurements provided estimates for the key parameters of Toddler dynamics, which we used to make predictions on spatiotemporal dynamics of mesoderm migration.

We simulated wild-type migration in the presence or absence of the mesodermal Toddler sink. The simulation predicted that animal pole–directed migration of mesodermal cells would be abolished in the absence of the sink function if the Toddler gradient was absent (ubiquitous Toddler expression or *toddler^−/−^*) or reversed (preferential Toddler expression at the margin from the YSL; Fig. 4D). With sink function in mesodermal cells, the dynamics depended on one rescaled parameter, namely, the product of mesodermal Toddler consumption rate (α) and the coupling between the local slope of the Toddler gradient and the corresponding active mesodermal migration speed (β) (compare fig. S4, A and C; see Supplementary Materials). Introduction of this parameter resulted in local depletion of Toddler at the margin, where mesodermal cells internalize, thus forming a Toddler concentration gradient (Fig. 4, E and F). The resulting animal pole–directed migration of mesodermal cells sustains the Toddler gradient, with stronger sink function resulting in proportionally faster migration and higher independence of the location of the Toddler source. Thus, in agreement with our experimental findings, the self-generated gradient model suggests that mesodermal cells can shape a gradient from uniform Toddler concentrations (or even from an inverse Toddler gradient, as long as the sink function is strong enough) to direct their migration to the animal pole (Fig. 4E). The resulting Toddler gradient can be sustained even close to the margin (far from the migratory leading edge; Fig. 4F) because of the continuous production of Toddler.

This model further predicts a distinct pattern of velocities across mesodermal cells, in which cell velocities increase as a function of the distance from the margin but decrease overall as a function of time (Fig. 4G). To test this prediction experimentally, we used light sheet microscopy to image transgenic zebrafish embryos expressing *drl:GFP* (*draculin* promoter driving GFP expression), which specifically labels ventrolateral mesoderm during gastrulation (*12*, *28*), injected with *h2b-RFP* mRNA to label all nuclei. This reporter allowed us to identify and track ventrolateral mesodermal cells and their progenitors (see Supplementary Materials for details). We found that the experimentally measured average mesodermal cell velocity was indeed highest at the leading edge of the mesodermal cells and decreased overall with the progression of gastrulation (Fig. 4H). Therefore, with a single fitting parameter (strength of self-generated directionality as mentioned above; see Supplementary Materials for details), theoretical predictions could recapitulate the experimental kymographs (Fig. 4H). Moreover, the shape of the mesodermal cell density profile along the animal-vegetal axis in wild-type and *toddler^−/−^* embryos predicted by the model (fig. S4H) was also consistent with experimental data (fig. S4, I and J).

Next, we simulated mesodermal migration dynamics in the absence of Toddler. In this case, the only mode of mesodermal cell movements is random cell motility (with coefficient *D_c_* estimated from short-term measurements of transplanted cell displacements in *toddler*^−/−^ embryos; see fig. S4, K and L). The model predicted that mesodermal cells migrate approximately half the distance in *toddler^−/−^* embryos compared to that in wild-type embryos, and that animal pole–directed migration rapidly decreased over time (Fig. 4, G and H), as expected from a random migration process. We found that the migration front of mesodermal cells in *toddler^−/−^ tg(drl:GFP)* embryos did not progress as far as in wild-type *tg(drl:GFP)* embryos and had nearly zero net velocity 4 hours after the onset of internalization (Fig. 4H). In summary, our theoretical predictions, supported by experimental measurements in wild-type and *toddler^−/−^* embryos, strongly suggest that mesodermal cells are being guided to the animal pole by a self-generated Toddler gradient.

### Aplnr-mediated removal of Toddler at the margin self-generates a Toddler gradient

Our hypothesis that mesodermal cells self-generate a Toddler gradient that guides their own migration to the animal pole makes four predictions: First, mesodermal cells are required to locally remove Toddler and thereby mediate the directed migration of individual cells to the animal pole; second, there is a maximal threshold of ligand concentration the system can compensate for; third, Toddler levels in wild-type embryos are highest at the animal pole and lowest at the margin; and fourth, the animal pole-directed migration of mesodermal cells is a phenomenon of collective rather than individual cell migration.

**Fig. 5.**
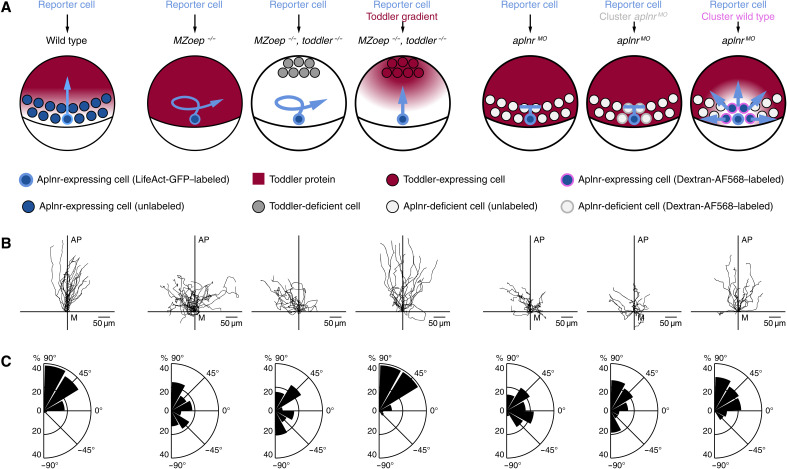
Aplnr-expressing mesodermal cells are required to establish a Toddler gradient. Cell transplantation assays to test for the necessity of Aplnr-expressing mesodermal cells as a Toddler sink. Transplanted LifeAct-GFP–labeled wild-type reporter cells were used as a read-out for the presence of a Toddler gradient. (**A**) Schematic representation of scenarios tested. From left to right: (i) Transplantation of reporter cells into a wild-type host embryo (*n* = 21). (ii) Transplantation of reporter cells into an *MZoep^−/−^* host embryo (*n* = 22). (iii) Transplantation of reporter cells into an *MZoep^−/−^*, *toddler^−/−^* double-mutant host embryo. Additional transplantation of Dextran–Alexa Fluor 568–labeled control source cells to the animal pole (*n* = 16). (iv) Transplantation of reporter cells into an *MZoep^−/−^*, *toddler^−/−^* double-mutant host embryo. Additional transplantation of Toddler-expressing source cells to the animal pole (*n* = 15). (v) Transplantation of reporter cells into an *aplnr ^MO^* embryo (*n* = 20). (vi) Cotransplantation of one to five reporter cells and a large number of Dextran–Alexa Fluor 568–labeled Aplnr-deficient control cells into *aplnr^MO^* host embryo (*n* = 15). (vii) Cotransplantation of one to five reporter cells and a large number of Aplnr-expressing cells into *aplnr^MO^* host embryos (*n* = 16). (**B**) Tracks of transplanted reporter cells [order as described in (A)]. Cells were tracked for 90 min after internalization. *x* axis = margin; *y* axis = animal-vegetal axis; coordinate origin = start of track. (**C**) Rose plots showing relative enrichments of orientations of polarity. 90° = animal pole; 0° = ventral/dorsal; −90° = vegetal pole.

To test the requirement of mesodermal cells as a sink, we transplanted LifeAct-GFP–labeled cells from the margin of a wild-type embryo to the margin of stage-matched host embryos that either have (wild type) or lack (*MZoep^−/−^*) the proposed mesodermal sink (Fig. 5A). The reporter cells showed directional migration in the wild-type host but lost their directionality and polarity in the *MZoep^−/−^* host (Fig. 5, B to D; fig. S5, A to D; and movie S9), where they formed multiple actin-rich protrusions in various directions (Fig. 5C; fig. S5, A to D; and movie S9), in line with excessive chemokine stimulation from all directions (*29*). To confirm that the absence of a Toddler gradient causes this loss of directional migration, we engineered a Toddler gradient in *MZoep^−/−^*, *toddler^−/−^* double mutant embryos by transplanting a cluster of Toddler-expressing cells to the animal pole to establish a local source of Toddler (Fig. 5A). This ectopically induced Toddler gradient was indeed able to rescue directional polarization, protrusion formation, and migration of wild-type reporter cells toward the animal pole in embryos lacking mesendodermal cells (Fig. 5, B to D; fig. S5, A to D; and movie S9). Therefore, mesendodermal cells are required to establish a Toddler gradient, which guides them toward the animal pole.

Although only a few examples of self-generated gradients have been described, different mechanisms have been observed to mediate their formation, such as enzymatic chemokine digestion at the cell surface (*22*) or chemokine internalization via a scavenger receptor (*20*, *21*). It has previously been reported that Toddler binds Aplnr-expressing cells (*11*) and induces Aplnr internalization (fig. S6, A to C) (*10*), which, based on the general mechanism of GPCR-ligand interaction and signaling (*30*), should lead to the concomitant endocytosis and degradation of Toddler, making Aplnr an excellent candidate for the local uptake and thus removal of Toddler. To test this possibility we transplanted individual LifeAct-GFP–labeled wild-type reporter cells to the margin of a sphere-stage *aplnr ^MO^* host embryo, which lacks Aplnr expression (Fig. 5A). The reporter cells lost their ability to polarize, lacked the typical animal pole-directed bias in protrusion formation, and failed to migrate toward the animal pole in the *aplnr ^MO^* host, phenocopying their behavior in the *MZoep^−/−^* host (Fig. 5, B and C; fig. S5, A to D; and movie S10). These results, together with the chemokine assays presented in Fig. 2, establish a dual role for Aplnr in mesodermal cells during zebrafish gastrulation as (i) a scavenger receptor to generate the Toddler gradient by removing it from the environment and (ii) a chemokine receptor to sense the Toddler gradient and direct cell migration.

Self-generated gradients are known to be robust to fluctuations in chemokine levels. However, the limited availability and turnover of the receptor predicts a maximal threshold of ligand concentration the system can compensate for and maintain the balance between ligand and receptor. In light of the dual role of Aplnr, we expanded the computational model described above to account for the Aplnr turnover during Toddler removal. Because of the finite capacity of Aplnr to remove Toddler, the model predicts that, above a threshold level of Toddler, mesodermal migration would become nondirectional, resembling *toddler^−/−^* embryos (fig. S7A). It further predicts that these migration defects at high Toddler levels can be rescued by simultaneous overexpression of Aplnr (fig. S7B). We confirmed both of these predictions experimentally. Overexpression of Toddler in wild-type embryos caused mesodermal mis-migration phenotypes mimicking *toddler^−/−^* embryos (fig. S7C), as previously observed (*10*). However, simultaneously increasing the number of mesodermal cells (and thereby the level of Aplnr expression in these embryos) by blocking the expression of the Nodal inhibitor Lefty2 (*31*) restored mesoderm migration in embryos with intermediate levels of Toddler overexpression (5 pg of *toddler* mRNA; fig. S7C). Together, these observations provide independent evidence for Aplnr acting as a scavenger receptor for Toddler and support the model of a self-generated gradient.

**Fig. 6.**
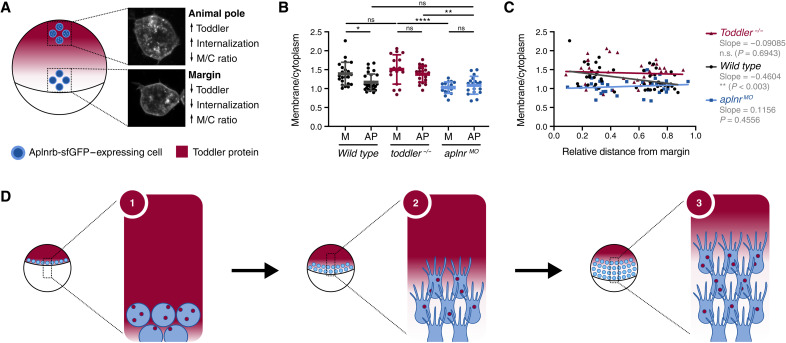
Aplnrb-sfGFP internalization allows detection of a Toddler concentration gradient. (**A**) Schematic representation of transplantation assay in a wild-type host embryo. Transplantation of Aplnrb-sfGFP–expressing *toddler^−/−^* cells to the animal pole and margin of the host embryo. Insets display representative confocal images of transplanted cells at animal pole and margin in a wild-type host embryo. (**B**) Quantification Aplnrb-sfGFP internalization levels of marginal (M) and animal pole (AP)–located transplanted cells in wild-type [*n*(M) = 22, *n*(AP) = 29], *toddler^−/−^* [*n*(M) = 16, *n*(AP) = 21], or *aplnr^MO^* [*n*(M) = 17, *n*(AP) = 18] host embryos, as determined by the ratio of membrane to cytoplasm fluorescence intensity. (**C**) Ratio of membrane to cytoplasm fluorescence intensity of transplanted cells relative to their distance to the margin. *P* value indicates significance of regression being different to 0 as determined by *F* test. (**D**) Schematic representation of the self-generated Toddler gradient: (i) Toddler is ubiquitously expressed throughout the embryo cap. Mesodermal progenitors internalize at the margin and express Aplnr, which acts as a scavenger receptor for Toddler. (ii) Aplnr binds and internalizes Toddler, which generates a local Toddler concentration gradient in front of the mesodermal cells, providing a directional cue. (iii) Aplnr also acts as a chemokine receptor to sense the self-generated Toddler guidance cue and induces the directed migration of mesodermal cells toward the animal pole, while continuously internalizing Toddler and shaping the local gradient. Data are means ± SD. Significance was determined using one-way ANOVA with multiple comparison; ****P < 0.0001; **P < 0.01; *P < 0.05.

Our model of a self-generated gradient predicts Toddler levels to be high at the animal pole and low at the margin because of the local uptake through Aplnr-expressing mesodermal cells. To experimentally confirm this prediction, we aimed at detecting local differences in Toddler levels. Because fluorescently or multi-epitope tagged Toddler is not functional, and because available Toddler antibodies (*11*) are not sensitive enough to detect endogenous Toddler, a direct visualization of the Toddler gradient is currently not feasible, prompting us to use Aplnr internalization (fig. S6) as a read-out for the local Toddler concentration. To establish the feasibility of this approach, we first determined the maximal difference that could be expected between the animal pole and margin in wild-type embryos. We assessed maximal and minimal Aplnr internalization levels by measuring the ratio of membrane-localized versus internalized Aplnr in transplanted Aplnrb-GFP–expressing *toddler^−/−^* cells in the presence of uniformly high Toddler levels (*aplnr*^MO^ host embryos) and in the absence of Toddler (*toddler*^−/−^ host embryos; Fig. 6, A to C, and fig. S6, D to G). This revealed a relatively small yet significant increase in Aplnr-GFP internalization in *aplnr*^MO^ versus *toddler*^−/−^ host embryos. To confirm the presence of a Toddler gradient in wild-type embryos, we transplanted Aplnrb-GFP–expressing cells from a sphere-stage *toddler*^−/−^ host embryo either to margin or to the animal pole at the ventral side of a shield-stage wild-type host embryo. Consistent with higher levels of Toddler at the animal pole, the level of Aplnr internalization was increased at the animal pole compared to the margin (Fig. 6, A to C, and fig. S6, D to G). This local difference in Toddler levels between the animal pole and margin was not detected in either *toddler*^−/−^ or *aplnr ^MO^* host embryos (Fig. 6, A to C, and fig. S6, D to G). Together, these results confirm the existence of a Toddler gradient that depends on the sink activity of Aplnr-expressing mesodermal cells.

Last, a critical number of cells is required to establish a self-generated gradient. Accordingly, the formation of the Toddler gradient is predicted to require a sufficient number of cells to locally remove enough chemokine (*32*), as seen by a decrease of animal pole–directed migration in simulations with a reduced number of mesodermal cells (fig. S5E). Therefore, we hypothesized that a cluster of Aplnr-expressing cells, but not a single Aplnr-expressing cell, would undergo directional migration in a uniform Toddler environment when transplanted to the margin. To test this hypothesis, we assessed the migratory ability of a small number (up to 10) of LifeAct-GFP–labeled wild-type reporter cells cotransplanted with a large number (more than 50) of Dextran–Alexa 568–labeled, Aplnr-expressing (sink^+^), or Aplnr-lacking (sink^−^) cells, to the margin of an *aplnr ^MO^* host embryo (Fig. 5A). The reporter cells migrated randomly and failed to reach the animal pole when cotransplanted with cells that lacked Aplnr (Fig. 5, B and C; fig. S5, A to C; and movie S10). However, when the cotransplanted cells expressed Aplnr, the reporter cells displayed increased track straightness, reduced ectopic protrusion formation and increased directional, radial outward-directed polarization (Fig. 5, B and C; fig. S5, A to C; and movie S10), indicating the presence of a guidance cue. In summary, these results provide evidence that a cluster of Aplnr-expressing cells is sufficient to restore sink activity and directional migration of reporter cells, in line with the model of self-generated gradients.

## DISCUSSION

This study describes a previously unknown, self-generated Toddler gradient that is formed and sensed by a single molecular mediator, the Apelin receptor, to guide the animal pole–directed migration of ventrolateral mesoderm during zebrafish gastrulation (Fig. 6D). Through the global production and local degradation of Toddler, the location of the mesoderm sink defines the directionality of cell migration toward the animal pole, as it both generates and senses a Toddler gradient that is self-sustained and inversely proportional to the mesodermal cell density (Fig. 6D).

Different types of self-generated gradients have recently been discovered as powerful guidance mechanisms (*20*–*22*, *33*). Self-generated chemokine gradients are characterized by one shared feature: Both the function of chemokine gradient formation and sensing are found within the migrating unit at opposite poles of the tissue (*20*, *21*) or even the same cell, as suggested by theoretical work and studies with cultured cells in vitro (*22*). Additional studies based on computational modeling (*34*, *35*) or in vitro experiments (*36*) have further suggested that removal of the chemokine and sensing of the gradient could be mediated by the same receptor. We provide direct experimental in vivo evidence that a GPCR, namely, the Aplnr, indeed executes both scavenger and sensor roles, acting as the sole molecular player in generating and sensing the directional cue that guides mesodermal cells to the animal pole. A conceptually similar finding, namely, that signaling GPCRs modulate extracellular guidance cues, has been reported for immune cell migration while this study was under review (*37*). The chemokine receptor CCR7 was shown to both sense and self-generate functional CCL19 chemokine gradients in vitro, thereby collectively organizing the comigration of several immune cell types. Together with our study, this highlights the potentially broadly applicable concept of GPCR-mediated self-generated signal gradients in diverse biological contexts.

The use of a self-generated gradient, in particular those based on a single receptor, holds several advantages over a preexisting gradient for the migration of a dynamic tissue, like the arising mesoderm. First, self-generated gradients can robustly act over long distances (*32*), which is necessary for mesodermal cell migration, with the space from margin to animal pole spanning more than 500 μm during zebrafish gastrulation. Second, the simple two-component system (here: Aplnr and Toddler) of a single receptor–based self-generated gradient can be easily adjusted to a dynamically changing tissue and thus circumvents the need to establish stable domains with distinct functions. Instead, the shape of the gradient is determined by the position of the migrating cell front rather than the total size of the tissue. This is particularly beneficial during gastrulation, as the continuous internalization of mesodermal cells and the vegetal pole–directed movement of the margin causes a steady increase in cell number and expansion of tissue size. Last, while preexisting gradients require tight regulation of steepness and chemokine concentration along the gradient to ensure reliable cell guidance, self-generated gradients can compensate for changes in length scale (as described above) and chemokine levels by adjusting the rate of chemokine breakdown (*38*). Therefore, self-generated gradients may have evolved to accommodate different architectures of complex migrating tissues by providing the necessary flexibility to adjust to changing environments and migration modes.

Our computational simulations and experimental analyses of mesodermal cell migration patterns are consistent with the formation of an Aplnr-dependent Toddler gradient in wild-type embryos because in the absence of Toddler as a guidance cue, mesodermal cell migration was mostly driven by random cell motility. However, it is important to note that *toddler^−/−^* cells retained some residual bias for migration toward the animal pole (Figs. 1, 4, and 5). These findings suggest that—at least in the absence of a Toddler-based guidance cue—migration is aided by additional, Toddler-independent mechanisms, which could include contact inhibition of locomotion (*39*, *40*) or biomechanical forces (*41*, *42*).

Morphogenetic movements during gastrulation across the animal kingdom share common principles. In amniotes, including mouse and human, mesodermal progenitors internalize at the primitive streak before migrating anteriorly as a noncoherent cell sheet (*1*). Previous studies have shown that, while conserved, Toddler and Apelin, the other ligand of Aplnr, are not essential for gastrulation movements in mice (*43*). However, based on the presented advantages and similar nature of morphogenetic movements across species, we hypothesize that self-generated gradients of different or redundant chemokine receptor-ligand pairs could present a universal mechanism underlying the anterior migration of mesodermal progenitor cells in gastrulation, as well as other cell migration events throughout development and physiology.

## MATERIALS AND METHODS

### Ethical statement

All fish experiments were conducted according to Austrian and European guidelines for animal research and approved by the Amt der Wiener Landesregierung, Magistratsabteilung 58 - Wasserrecht (animal protocols GZ: 342445/2016/12 and MA 58-221180-2021-16).

### Zebrafish husbandry

Zebrafish (*Danio rerio*) were raised according to standard protocols (28°C water temperature, 14-hour light/10-hour dark cycle). TLAB fish were generated by crossing AB and natural variant TL (Tupfel Longfin) zebrafish and used as wild type for all experiments. *MZoep^−/−^* (*26*) and *toddler^−/−^* (*10*) double mutant, as well as *tg(drl:gfp)* (*28*), *toddler^−/−^* lines, and *tg(gsc:CAAX-GFP)* (*44*) wild-type lines were generated by crossing the two respective lines.

### Injection of mRNAs and morpholinos into zebrafish embryos

Capped mRNAs for *toddler*, *oep*, *aplnrb*, *aplnrb-sfgfp*, *lifeact-gfp*, *human h2b-bfp*, *human h2b-rfp*, and *f’bfp* were transcribed from linearized plasmids using the SP6, T7, or T3 mMessage Machine Kit (Ambion), according to the manufacturer’s protocol. All mRNAs were injected into one-cell stage embryos unless indicated otherwise. All plasmids were previously described (table S1).

Double knockdown of *aplnra* and *aplnrb* and knockdown of *lefty2* were performed using morpholino injection as previously described (*10*, *45*–*48*). Briefly, 0.5 and 1 ng of MOs against *aplnra* (cggtgtattccggcgttggctccat; GeneTools) and *aplnrb* (cagagaagttgtttgtcatgtgctc; GeneTools), respectively, or 12.5 ng of MO against *lefty2* (agctggatgaacagagccatgac; GeneTools) were injected into one-cell stage embryos. A control MO (cctcttacctcagttacaatttata; GeneTools) was used at equivalent concentrations.

### Live cell imaging

#### Light sheet microscopy

For light sheet microscopy, four embryos were mounted in 0.6% low-melt agarose in 1× phosphate-buffered saline (PBS) in glass capillaries (Brand, 20 μl). The capillary was placed in the heated (27°C), fish water-filled sample chamber of a Zeiss Z1 light sheet microscope. The two most suitably oriented embryos were imaged.

To assess morphology and migration behavior of wild-type and *toddler^−/−^* cells, four successfully transplanted embryos (only one genotype per experiment) were mounted. The two embryos that had the best orientation (animal pole straight up or down + lateral orientation + transplanted cells in the center of the frame at the margin) were selected for imaging. Time-lapse series of Z-stacks were taken from both embryos over 5 hours (from sphere stage to end of gastrulation) with a 20× objective (table S3).

For global cell tracking experiments, wild-type *tg(drl:gfp)* or *toddler^−/−^ tg(drl:gfp)* embryos were injected with 100 pg of *h2b-rfp* mRNA at the one-cell stage and mounted at sphere stage. The 0.6% low-melt agarose was supplemented with fluorescent beads (1:1000; TetraSpeck microspheres, 0.2 μm, fluorescent blue/green/orange/dark red, Invitrogen no. T7280) that were used to adjust the light sheet offset and stabilize the imaging sequence. Four embryos were mounted, and the two most ideally oriented embryos (animal pole up or down) were selected for imaging. Time-lapse series of Z-stacks were taken from both embryos over 8 hours with a 10× objective (table S3). At 24 hours post fertilization, z-stacks from four positions around the embryo were acquired (0°, 90°, 180°, and 270° in reference to the position of the time course imaging) to determine the location of dorsal and ventral sides within the embryo. Last, six z-stacks of 1000 z-slices were acquired with lasers turned off (used to remove background during subsequent data analysis). Raw light sheet data were converted to tiff files. For cell morphology analyses, the data were binned 4× in *xy* to allow for better data handling. The region of interest (internalizing migrating cell) was identified in binned images and automatically cropped (in *x*/*y*/*z* and time) in the raw data, using a custom-made app (https://drive.google.com/drive/folders/1183kxnarTEyb1-4Otzyh0RlQhEnHO8Oo?usp=sharing). Cropped imaging files were analyzed as described below.

#### Confocal microscopy

For confocal imaging, embryos were mounted in the desired orientation in a drop of 0.8% low-melt agarose in 1× PBS on round glass bottom dishes (ibidi). Agarose drops were left to harden before dishes were filled with E3 medium (5 mM NaCl, 0.17 mM KCl, 0.33 mM CaCl_2_, 0.33 mM MgSO_4_, 10^−5^% methylene blue). Time-lapse movies of z-stacks were acquired on an inverted LSM800 Axio Observer (Zeiss) with temperature incubation (27°C) for 6 hours. To assess Aplnrb-GFP internalization, z-stacks for each embryo were taken for only one time point at the sphere stage (fig. S6, A to C) or the shield stage (Fig. 4, E to G, and fig. S6, D to G).

### Transplantation assays

#### Cellular phenotype

To assess the cell migration behavior of individual cells after internalization, donor embryos were injected with 100 pg of *lifeact-gfp* mRNA. For light sheet imaging experiments only, host embryos were injected with 100 pg of *h2b-rfp* mRNA. Between one and five cells were taken from the marginal region of donor embryos at the sphere stage and transplanted to the margin of stage- and genotype-matched host embryos. At the dome stage, embryos were mounted laterally with transplanted cells facing the glass dish and imaged on a confocal microscope or mounted in a glass capillary for light sheet microscopy and imaged as described above. For light sheet microscopy, individual frames were acquired in 1-min intervals, while for confocal microscopy, individual frames were acquired in 5-min intervals. Analysis of the cellular phenotype (see below for details) confirmed that, except for slight differences in cell speed, results were consistent between light sheet and confocal data. The differences in cell speed likely stem from the lower time resolution in confocal imaging that cannot fully resolve all fluctuations in the migratory tracks and, therefore, might lead to an underestimation of the actual distance traveled by the cell in 5 min.

#### Cell autonomy

To assess the cell autonomy of Toddler signaling, embryos were prepared, and transplantations were performed as described above. Donor and host embryos were stage-matched but of opposite genotypes (transplantation of wild type into *toddler^−/−^* and vice versa). Imaging and subsequent analysis were performed blindly.

#### Chemokine assay

All embryos used for the chemokine assay were *toddler^−/−^*. Donor^receptor^ embryos were injected at the one-cell stage with 150 pg of *lifeact-rfp* mRNA only (control) or in combination with 200 pg of *aplnrb-sfGFP* mRNA. Donor^ligand^ embryos were injected at the one-cell stage with 100 pg of Dextran–Alexa Fluor 488 only (control) or in combination with 100 pg of *toddler* mRNA. Host embryos were injected at the one-cell stage with 150 pg of *h2b-bfp* mRNA. Embryos were left to develop until sphere stage. A total of 50 to 100 cells were taken from donor^ligand^ embryos and transplanted to the animal pole of stage-matched host embryos. One to ten cells were taken from donor^receptor^ embryos and transplanted to the host embryo at positions surrounding the animal pole. Embryos were mounted on the animal pole and imaged as described above.

#### Localized Toddler source

Animal pole expression of Toddler was achieved by transplantation assays. Donor embryos were injected at the one-cell stage with 100 pg of Dextrane–Alexa Fluor 488 only (controls) or in combination with 200 pg of *toddler* mRNA. Dextrane–Alexa Fluor 488 was used to trace successful injection and transplantation. At the sphere stage, 50 to 100 cells were taken from donor embryos and transplanted to the animal pole of stage-matched *toddler^−/−^* host embryos. For uniform expression of Toddler, 100 pg of Dextran–Alexa Fluor 488 only (controls) or in combination with 2 pg of *toddler* mRNA was injected into one-cell stage *toddler^−/−^* embryos. To achieve marginal expression, 100 pg of Dextrane–Alexa Fluor 488 only (controls) or in combination with 10 pg of *toddler* mRNA was injected into the YSL of 1k-cell stage embryos. Embryos were collected for in situ hybridization (see below) at 75% epiboly.

#### Sink removal

To test for sink function of mesendodermal cells and scavenger function of Aplnr, wild-type donor embryos were injected with 100 pg of *lifeact-gfp* mRNA at the one-cell stage. Host embryos were either untreated (*MZoep^−/−^*) or injected with *aplnr* MOs to inhibit *aplnr* mRNA translation in wild-type embryos. One to five donor cells were taken from the margin at sphere stage and transplanted to the margin of a stage-matched host embryo. Transplantations of more than 10 cells were excluded from analysis to avoid the possibility of sink reintroduction. Embryos were mounted laterally with transplanted cells facing the objective and imaged using confocal microscopy as described above.

#### Toddler gradient

To assess the sufficiency of a Toddler gradient to guide cell migration in the absence of mesendodermal cells, transplantations were performed using *MZoep^−/−^, toddler^−/−^* double-mutant host embryos. Wild-type donor^reporter^ embryos were injected with 100 pg of *lifeact-GFP* mRNA at the one-cell stage. *MZoep^−/−^*, *toddler^−/−^* double-mutant donor^source^ embryos were injected at the one-cell stage with 200 pg of Dextran–Alexa Fluor 568 only (controls) or in combination with 200 pg of *toddler* mRNA. A total of 50 to 100 donor^source^ cells were transplanted to the animal pole of a sphere stage host embryo. Subsequently, one to five LifeAct-GFP–positive donor^reporter^ cells were transplanted to the margin of the same host. Embryos were mounted laterally with transplanted cells facing the objective and imaged using confocal microscopy as described above.

#### Single versus collective cell migration

To generate a sink of Aplnr-expressing cells by transplantation, donor^reporter^ wild-type embryos were injected with 100 pg of *lifeact-gfp* mRNA at the one-cell stage. For donor^sink^, *tg(gsc:caax-gfp)* embryos were injected with 100 pg of Dextran–Alexa Fluor 568 at the one-cell stage. These embryos were used because they allow the identification and exclusion of the dorsal region at transplantation. Host and donor^sink^ embryos were injected with 0.5 ng of *aplnra* MO and 1 ng of *aplnrb* MO. At the late dome stage, 50 cells from the ventral margin of donor^sink^ embryos followed by 5 to 10 cells from the margin of donor^reporter^ embryos were taken into the same needle and transplanted to the margin of stage-matched host embryos. Embryos were mounted laterally with transplanted cells facing the objective and imaged as described above. Imaging and subsequent analysis were performed blindly.

#### Detection of Toddler gradient through Aplnrb-sfGFP internalization levels

To assess Aplnrb-sfGFP internalization levels depending on the cell position along the animal-vegetal axis within the embryo, host embryos (wild type or *toddler^−/−^*) were either left untreated or injected with 0.5 ng of *aplnra* MO and 1 ng of *aplnrb* MO, as indicated. Donor *toddler^−/−^* embryos were laid 2 hours after host embryos and injected with 200 pg of Aplnrb-sfGFP mRNA and 200 pg of Dextrane. Once the host embryos reached the shield stage, up to 20 cells were transplanted from the sphere-staged donor embryo to two positions within a single host embryo, the ventral margin and the animal pole. Embryos were left to recover for 15 min before being mounted laterally in 0.8% low-melt agarose with transplanted cells facing the objective and imaged as described above.

### Imaging analysis

#### Protrusions and polarity

Cells of interest for analysis were identified in the time-lapse movies based on several criteria:

1) Ventral or lateral position in the host embryo: Cells that were transplanted to the dorsal side (as indicated by visible convergence and extension movements as well as the formation of somites at the end of the time lapse) were excluded from the analysis.

2) Only internalized cells were analyzed. Internalization was identified by a cell’s position in the *z*-dimension (lower cell layer) and a change in direction that indicates the transition from vegetally directed epiblast to animally directed hypoblast cell movement.

3) Sufficiently long cell tracks: Analysis of a cell had to be possible for at least 15 (light sheet; ≙30 min) or 12 (confocal; ≙1 hour) time frames. Every cell that divided, moved out of frame, or overlapped with another transplanted cell within this time window was excluded from analysis.

4) Analysis was performed before ~70% epiboly (before the onset of convergence and extension movements).

Protrusions were distinguished based on the LifeAct-GFP signal of polymerized actin and defined by the following characteristics:

1) Lamellipodia: actin-mesh that expands several micrometers in width; wide protrusion that extends beyond the cell body.

2) Filopodia: thin actin strings that extend from the cell body.

3) Bleb: actin-free (marked by cytoplasmic, dispersed LifeAct-GFP), round membrane extension with polymerized actin at its base.

Each cell was analyzed from the time of internalization until one of the four criteria above was no longer applicable. In some cases, a cell divided shortly after internalization; therefore, analysis was started 10 (light sheet) or 2 (confocal) time frames (≙10 min) after division to exclude mitosis-induced cell rounding and loss of polarity. Length and angle (in reference to animal pole) of protrusions were measured in ImageJ. A straight line through the center of the protrusion was drawn from the base (border to cell body) to the tip of the protrusion. A cell was counted as polarized if it was elongated and actin polymerization and protrusion formation were restricted to one side only, with additional protrusions (filopodia only) allowed within a 45° angle to either side. Protrusions were measured and counted for every cell in each time frame. Protrusion rate (protrusion/min) was calculated by normalizing the sum of all protrusions for one cell to the length of the time course (for light sheet) or the number of time frames (for confocal). To quantify orientation of polarization and protrusions, the numbers of angles within every 30° were counted and normalized to the total number of the same protrusion/polarity within the same genotype. As the position of dorsal and ventral in these embryos was not specifically determined, the left and right side of the resulting 360° rose plot were combined to be collectively counted as dorsal/ventral orientation.

#### Cell tracks

Cell tracks were obtained from maximum intensity projections of the cell of interest using tracking tools in Imaris (Imaris x64 9.7.2). First, time-lapse movies were corrected for epiboly movements to represent the cell movement relative to epiboly in the final cell tracks. In all confocal imaging datasets except for the Toddler/Aplnr chemokine assay (Fig. 2 and fig. S3), epiboly movement was determined by manually tracking the vegetal pole–directed progression of the margin in the bright-field images. The cell tracks were then corrected based on the resulting “epiboly track.” In the light sheet datasets (fig. S1) and the chemokine assay (Fig. 2 and fig. S3), epiboly movements were determined by automatically tracking (Imaris) H2B-RFP or H2B-BFP–labeled cell nuclei, respectively. The tracks of three to five nuclei that were successfully tracked throughout the entire time course were used as a reference for correction of the datasets. Cells of interest in all confocal data (Figs. 1, 2, and 5; and figs. S2, S3, S5, and S6) and light sheet data in Fig. 1 and fig. S1 were manually tracked. Final tracks were exported and assembled using Adobe Illustrator. Average speed was calculated by dividing track length by track duration. Track straightness was calculated by dividing displacement by track length. Net displacement to the source was determined by measuring the vertical distance between start and end point of the track. The end point of the track was either the point at which the cell first made contact with the source, or, if no contact was made, after 2 hours of imaging.

#### Global cell tracking

Global cell tracking of all nuclei in the acquired light sheet microscopy data (Fig. 4 and fig. S4) was based on the approach of Jaqaman *et al.* (*49*). To this end, detected nuclei were greedily interconnected into short tracklets, which were then merged to form the final tracks. The interconnection of both the detected nuclei and tracklets was computed by solving a linear assignment problem using LAPMOD, a sparse variant of the Jonker & Volgenant linear assignment solver (*50*). Very short tracklets of less than three time points were pruned. New tracklets were not allowed to form within a sphere with a radius of 10 μm. Maximum allowed displacement between consecutive time points was set to 10 μm. Gaps between tracklets no longer than three time points were closed if the maximum displacement per time point stayed below a threshold of 10 μm.

The nuclei were detected using a multiscale Laplacian of Gaussian filter (five scales between 1 and 2.5 μm) after preprocessing the imaging data using the following operations: (i) A per-pixel mean image was computed and used for background subtraction to simplify cell tracking; noise and outliers were removed below and above the 50th and 99.99th percentile of intensity. (ii) Working in the log domain, the volumes were downsampled 2× along *x* and *y* coordinates to obtain close-to-isotropic spacing; a 7 × 7 pixel median filter was applied for all *z* planes independently. (iii) Furthermore, to simplify the tracking, the sequence was stabilized using a translation transformation that was estimated at each time point from the locations of the beads around the embryo using RANSAC. In particular, aligning was started at a reference point, and bead locations were always prealigned at the current time point using past transformation; the bead locations fed into RANSAC estimation were selected as mutual nearest neighbors after this prealignment.

The global cell tracking was implemented in Python (https://drive.google.com/drive/folders/12Q36RQGpzItsEP4bC3OKYiWuM53ZC7sv?usp=sharing) using OpenCV, scikit-image, and scikit-learn (*51*, *52*). The tracker was optimized to take advantage of a SLURM-based computation cluster; however, the implementation also allows running locally on a single computer, but the runtime might be prohibitive.

For visualization and analysis of the trajectories (https://drive.google.com/drive/folders/1GRgfTBL03aOvJ6FsMxBItVcMB6h2_cZc?usp=sharing), a sphere was fitted to the extracted nuclei locations (*53*), with latitude and longitude coordinates and the radial distance from the center of the embryo. The reference sphere was aligned with the animal and vegetal pole matching the north and south pole, respectively. Afterwards, the animal-vegetal axis and the dorsoventral axis were interactively recovered. This brought all the experiments into a common reference frame and allowed for simpler interpretation.

Cell internalization was determined by calculating each cell’s frame-by-frame difference in radius at the margin. The minimum of the average changes indicated a peak of internalization movements, which occurred between 30 and 50% epiboly in all datasets. This was used to align the recordings in developmental timing.

To isolate the tracks of mesodermal cells, nuclei colocalizing with the Drl:GFP signal were identified. In relation to the background fluorescence, a cell was considered Drl:GFP marker positive with a green fluorescence level higher than 96% of all measurements (*z* score 1.8) for at least 50 frames.

The animal-to-vegetal pole velocity was measured specifically for ventrolateral mesoderm cells. This was calculated by the change in distance relative to the margin over a moving window of 10 min (14 frames). The distance to margin is the difference in latitude between the position of the cell and the position of the margin at the corresponding time point.

### Detection of a Toddler gradient via Aplnrb-sfGFP internalization levels

Using Fiji, a mask was generated for each Aplnrb-sfGFP–positive cell, encompassing the entire cell area (including cytoplasm and membrane) by manually outlining the cells (mask^full^). Each mask was than reduced by 1.25 μm around the entire circumference. The resulting reduced mask represented the cytoplasmic area (mask^cyto^). Mask^cyto^ was then subtracted from mask^full^, resulting in a consistently 1.25-μm-thick band as a mask for the membrane (mask^membr^). The mean fluorescent intensity was measured within mask^membr^ and mask^cyto^, and the ratio (mask^membr^/mask^cyto^) was calculated as a proxy for Aplnrb-sfGFP internalization levels in each cell.

### Compensation of Toddler overexpression

Overexpression of Toddler was achieved at different levels by injecting 0 pg (water control), 5 pg, or 10 pg of *toddler* mRNA into one-cell stage wild-type embryos. Half of the embryos of each batch were additionally injected with *lefty2* MO to enhance Nodal signaling and thus increase the number of mesodermal cells. Embryos were collected for in situ hybridization at 75% epiboly (see below).

### In situ hybridization

In situ hybridization was performed as previously described (*54*). Briefly, wild-type and *toddler*^−/−^ embryos were fixed at 75% epiboly in 3.7% FA overnight and hybridized with a DIG-labeled antisense probe against *aplnrb* (*10*). After BCIP/NBT/Alkaline-phosphatase–staining, embryos were dehydrated in methanol and imaged in BB/BA on a Stemi 305 steromicroscope (Zeiss).

To quantify the mesoderm migration phenotype, embryos were imaged laterally at 75% epiboly. The spread of *aplnrb-*positive cells was measured in ImageJ on the ventral side and normalized to the distance between margin and animal pole to account for differences in epiboly progression between individual embryos. Data were normalized to the average spread in wild-type embryos.

To assess the mesodermal cell density profile of embryos, the gray values for a 100-pixel wide rectangle from animal pole to margin were measured using ImageJ and inverted (0 = white, 255 = black). Values for all 100 pixels in one row were averaged. The lowest value measured along the animal-margin axis was subtracted as background, and measurements were normalized to the highest value (= 1). All background-subtracted pixel values were plotted as position along the animal-margin axis (margin = 0, animal pole = 1).

### Internalization of Aplnr

To assess Aplnrb-GFP internalization in the absence of Toddler or upon Toddler overexpression (fig. S6, A to C), *toddler^−/−^* embryos were injected at the one-cell stage with 100 pg of *f’bfp* mRNA and 50 pg of *aplnrb-sfgfp* mRNA. At the 32-cell stage, 100 pg of Dextrane–Alexa Fluor 568 only (controls) or in combination with 100 pg of *toddler* mRNA was injected into a single blastomere. Embryos were left to develop until the sphere stage and then mounted on the animal pole and imaged as described above. To quantify Aplnrb-GFP internalization, cell outlines were manually traced in ImageJ based on the f’BFP signal. Mean fluorescence intensity of Aplnrb-GFP was measured for membrane and cytoplasm and multiplied with the membrane and cytoplasmic area, respectively. The ratio of total membrane and cytoplasmic signal was used as a measure of the internalization rate.

### Toddler half-life assessment

To measure the half-life time of Toddler protein, *MZoep^−/−^, toddler^−/−^* embryos were collected and left to develop until 30% epiboly. At 30% epiboly, 1 μg of Toddler peptide [GL Biochem: DKHGTKHDFLNLRRKYRRHN**C**PKKR**C**LPLHSRVPFP, cysteine residues in bold were cross-linked according to (*55*)] was injected into the cell cap. Ten cell caps were collected every 30 min for 3 hours through manual removal of yolk and flash-frozen in liquid nitrogen.

Western blotting was performed according to standard protocols. Briefly, embryo caps were supplemented with 10 μl of 2× Laemmli buffer and boiled for 5 min at 95°C. SDS–polyacrylamide gel electrophoresis was performed using Any kD Mini-PROTEAN TGX Precast Protein Gels (Bio-Rad), loading 10 embryo caps per lane. Blotting was performed using a wet-blot system (Bio-Rad). Protein detection was achieved using the following antibodies: anti-Toddler [rabbit, 1:500; provided by the Reversade Lab (*11*)] and anti-tubulin (mouse, 1:10,000; Sigma-Aldrich T6074).

## Supplementary Materials

### This PDF file includes:

Figs. S1 to S7 Tables S1 to S4 Supplementary information of computational modeling References
