# Supplementary material for: A self-generated Toddler gradient guides mesodermal cell migration
Source: Sci Adv. 2022 Sep 14;8(37):eadd2488. doi: 10.1126/sciadv.add2488 (PMC9473572; doi:10.1126/sciadv.add2488)
Supplement: Supplementary file 1 — Figs. S1 to S7 Tables S1 to S4 Supplementary information of computational modeling References [file sciadv.add2488_sm.pdf]

Science Advances
AAAS

Supplementary Materials for

A self-generated Toddler gradient guides mesodermal cell migration

Jessica Stock *et al.*

Corresponding author: Edouard Hannezo, edouard.hannezo@ist.ac.at; Andrea Pauli, andrea.pauli@imp.ac.at

Sci. Adv. **8**, eadd2488 (2022)
DOI: 10.1126/sciadv.add2488

The PDF file includes:

Figs. S1 to S7
Legends for movies S1 to S10
Tables S1 to S4
Supplementary information of computational modeling
References

Other Supplementary Material for this manuscript includes the following:

Movies S1 to S10

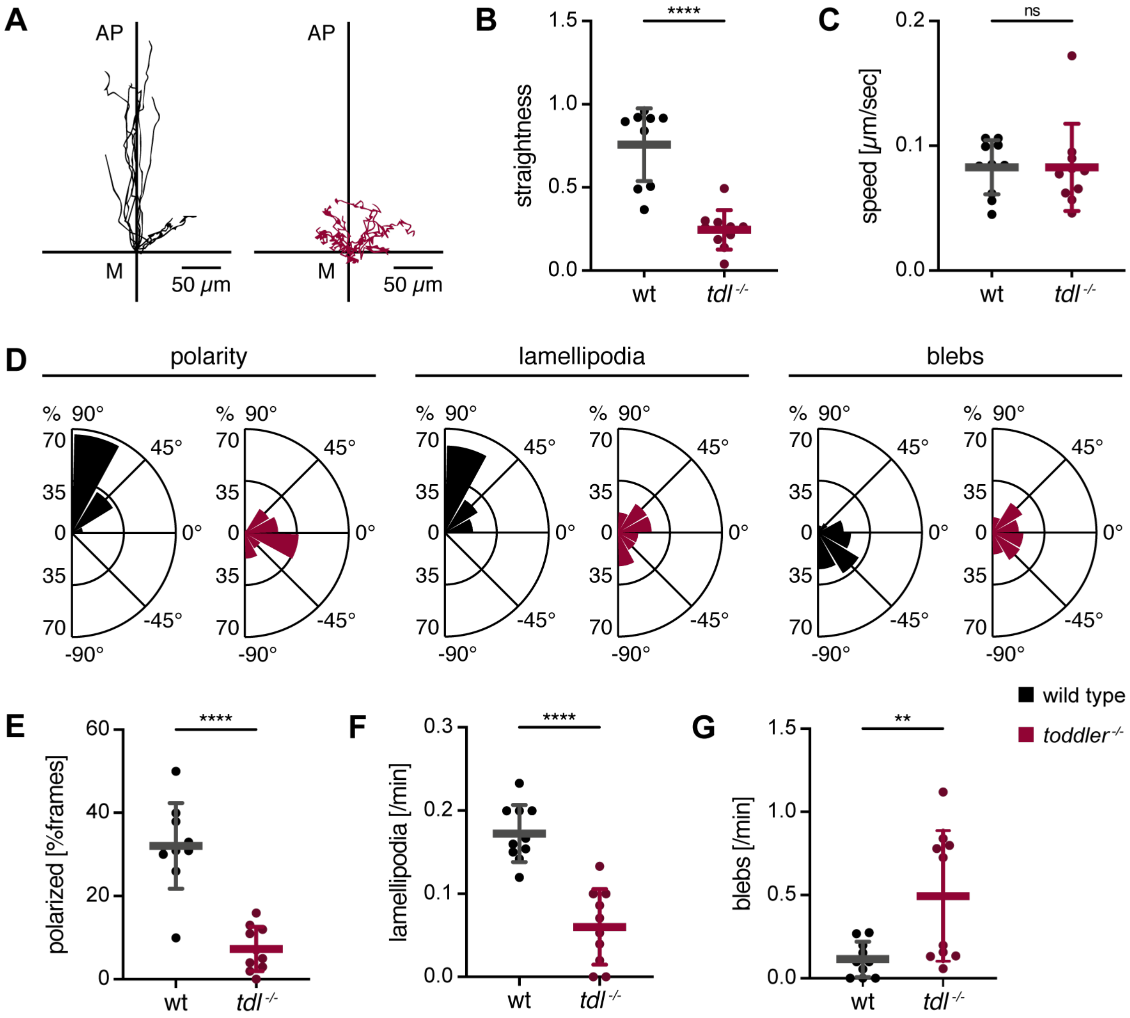
A
AP
M
50 μm
AP
M
50 μm
B
straightness
1.5
1.0
0.5
0.0
****
wt
tdl -/-
C
speed [μm/sec]
0.2
0.1
0.0
ns
wt
tdl -/-
D
polarity
lamellipodia
blebs
% 90°
70
35
0
45°
0°
-45°
-90°
E
polarized [%frames]
60
40
20
0
****
wt
tdl -/-
F
lamellipodia [/min]
0.3
0.2
0.1
0.0
****
wt
tdl -/-
G
blebs [/min]
1.5
1.0
0.5
0.0
**
wt
tdl -/-
wild type
toddler -/-

fig. S1. Mesendodermal progenitors are unpolarized and lack animal pole-directed protrusions in the absence of Toddler. Cell transplantation assays to assess the migration behavior of mesendodermal progenitors in the presence versus absence of Toddler signaling using light sheet microscopy. LifeAct-GFP-labelled reporter cells were transplanted from the margin of a wild-type or *toddler* $^{-/-}$ donor embryo to the margin of a stage- and genotype-matched host embryo. **(A)** Tracks of wild-type (left) and *toddler* $^{-/-}$ (right) reporter cells. Cells were tracked for 30 min after internalization. x-axis = margin; y-axis = animal-vegetal axis; coordinate origin = start of track. **(B)** Quantification of track straightness. **(C)** Quantification of migration speed. **(D)** Rose plots showing relative enrichments (percentages) of orientations of polarity, lamellipodia and blebs normalized to the total number of polarity axes or respective protrusions of all cells within the same genotype. **(E)** Quantification of cell polarity represented as the percentage of frames in which a cell was polarized. **(F)** Quantification of lamellipodia. Data represents newly formed lamellipodia per minute. **(G)** Quantification of blebs. Data represents newly formed blebs per minute. Data are means ± standard deviation (SD). Significance was determined using unpaired t test; ****, $p < 0.0001$; **, $p < 0.01$; n.s., not significant. n = 10 cells. Wild type (black); *toddler* $^{-/-}$ (red). Rose plots: 90° = animal pole; 0° = ventral/dorsal; -90° = vegetal pole. All images and graphs are oriented with the animal pole towards the top.

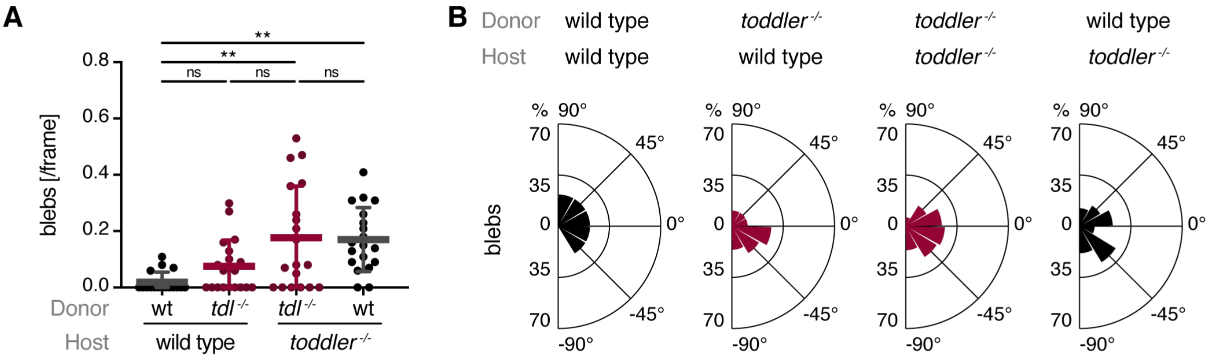

fig. S2. *toddler* $^{-/-}$ cells display an increase in cell blebbing. Cell transplantation assays to assess the cell autonomous or non-autonomous regulation of cell blebbing by Toddler signaling. **(A)** Quantification of blebs represented as the average number of blebs detected per frame (see Materials and Methods for classification of blebs). **(B)** Rose plots showing relative enrichments (percentages) of orientations of blebs, normalized to the total number of blebs of all cells within the same condition. Rose plots: 90° = animal pole; 0° = ventral/dorsal; -90° = vegetal pole. Data are means ± SD. Significance was determined using unpaired t test; **, $p < 0.01$; n.s., not significant.

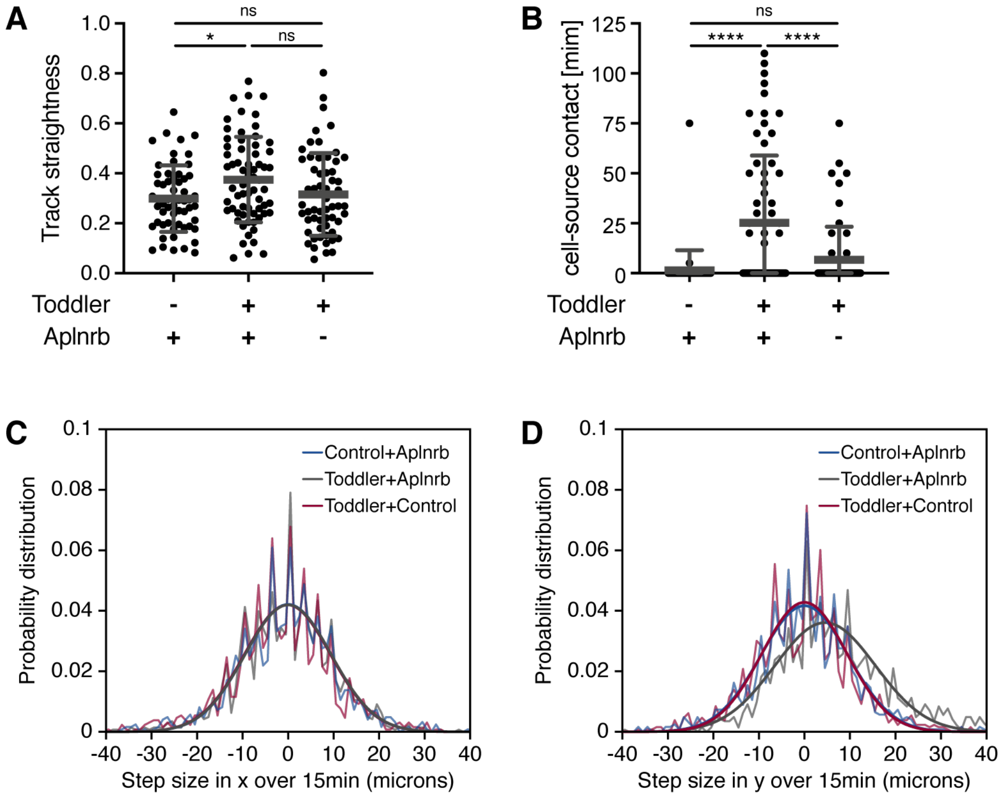

fig. S3. A localized source of Toddler attracts Aplnrb-sfGFP-expressing cells. Assessment of Toddler's ability to act as a chemoattractant for Aplnr-expressing mesodermal cells. **(A)** Track straightness of all cells imaged in Fig. 2B. Track straightness was calculated based on 120 min tracks as displacement divided by track length, irrespective of when the cells encountered the source. **(B)** Quantification of cell-source contact. The longest uninterupted time span, in which contact with a Toddler source cell was detected, was plotted for each cell. n = 56, 65 and 59 for the three different conditions, respectively. Data are means ± SD. Significance was determined using one-way ANOVA with multiple comparison; ****, $p < 0.0001$; *, $p < 0.05$; n.s., not significant. **(C-D)** Distribution of step size, defined as distance migrated by cells in the x- (C) and y- (D) directions within 15 minutes (x-direction: left-right movement in respect to the cell-source axis; y-direction: movement towards the source; see **Fig. 2A-C** for schematics and plotting of the trajectories), across all three conditions examined (Aplnrb-sfGFP-expressing cells migrating towards a Toddler-expressing cell, Aplnrb-sfGFP-expressing cells migrating towards a Toddler-negative control cell, Aplnrb-deficient control cells migrating towards a Toddler expressing source). All datasets are well-fitted by Gaussian distribution $P(s) = \frac{e^{-\frac{(s-s_0)^2}{2\sigma^2}}}{\sigma\sqrt{2}}$ (black lines), as predicted from a biased random walk, with the only non-zero bias s_0 occurring in the y-direction for the Aplnrb+Toddler condition. Best-fit variance: $\sigma = 9.50\ \mu m,\ 9{,}49\ \mu m,\ 9.47\ \mu m$ for resp. blue, grey and red datasets in panel C. Best-fit variance: $\sigma = 9.32\ \mu m,\ 11.05\ \mu m,\ 9.57\ \mu m$ for resp. blue, grey and red datasets in panel D, with a best-fit bias $s_0 = 4.55\ \mu m$ for the Toddler+Aplnrb condition.

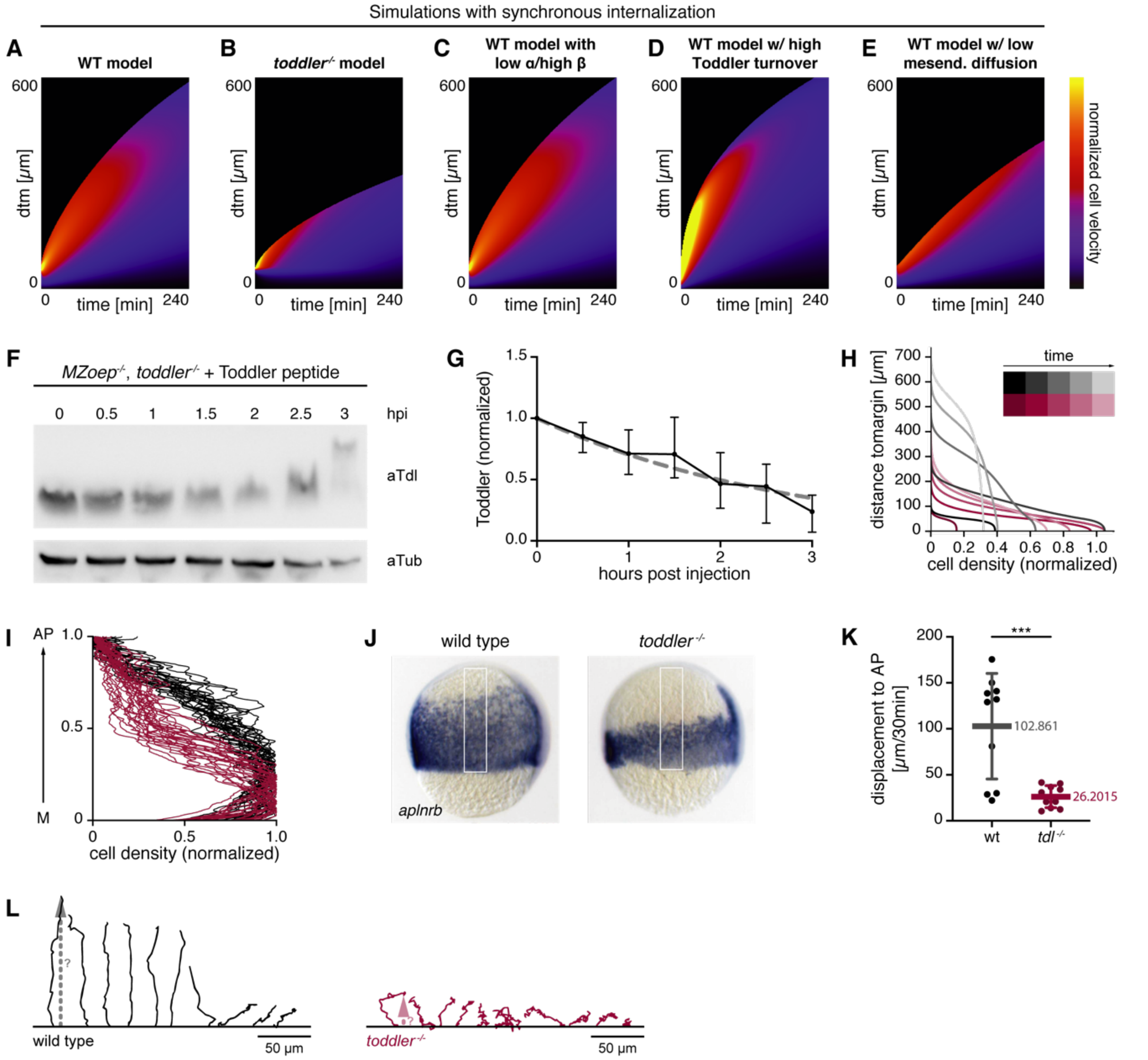

fig. S4. Determination of parameters for computational modeling. (A-E) Kymographs for normalized cellular velocities as a function of time (x-axis) and distance from the margin (dtm, y-axis): Sensitivity analysis of the model to different assumptions shown in the case of synchronous internalization from the onset (see Materials & Methods for details). **(A-B)** Simulations with the same simulation parameters as Fig. 4H for WT (**A**) and *toddler*$^{-/-}$ (**B**) embryos but for synchronous internalization, showing qualitatively similar dynamics. **(C)** Simulations keeping the product $\alpha\beta$ constant but multiplying β (coupling between local Toddler gradients and velocity) by 5 and dividing α (sink strength) by 5 gives rise to similar dynamics compared to panel (A). **(D)** Simulations with the same model parameters as (**A**) but with faster Toddler baseline degradation $\tau_T = 10s$, decreasing the range of Toddler gradient propagation. **(E)** Simulations with the same model parameters as (**A**) but with negligible mesendoderm random cell motility $D_m = 0$, giving rise to a smaller range of migration but qualitatively similar velocity profiles. **(F-G)** Assessing Toddler peptide stability in the presence and absence of Aplnr. **(F)** Western Blot analysis of the Toddler peptide degradation rate. *In vitro* synthesized Toddler peptide was injected into *MZoep*$^{-/-}$, *toddler*$^{-/-}$ double mutant embryos. Embryos were collected every 30 min for 3 hours and used for Western Blot analysis, probing for Toddler (aTdl) and alpha-Tubulin (aTub; loading control). **(G)** Quantification of Toddler levels (normalized to Tubulin) at different time points. Dotted line represents exponential fit for degradation curve that were used to calculate Toddler half-life. n=3. **(H-I)** Measurements of cell displacement towards the animal pole to determine Toddler-independent cell velocity in *toddler*$^{-/-}$ embryos. **(H)** Predicted spatiotemporal profile of mesoderm cell densities in the wild-type (grey) and *toddler*$^{-/-}$ (red) condition. **(I-J)** Experimental assessment of mesodermal cell density along the animal-margin axis. **(I)** Quantification of cell density along animal-margin axis in wild-type (black) and *toddler*$^{-/-}$ (red) embryos. n = 25 embryos. **(J)** Images for *in situ* hybridization for *aplnrb* of a representative wild-type (left) and *toddler*$^{-/-}$

(right) embryo. White box indicates area measured for quantification **(K)** Quantification of the net animal pole (AP)-directed displacement based on tracks in **(I)**. Data are means ± SD. Significance was determined using unpaired t test; ***, $p < 0.001$. **(L)** Individual migration tracks of cells presented in fig. S1. The perpendicular net displacement of each cell from the margin (dotted line) was measured using the end point of each cell track 30 min after internalization. Track start is at the margin. Animal pole is shown towards the top. Wild type (black), *toddler* $^{-/-}$ (red).

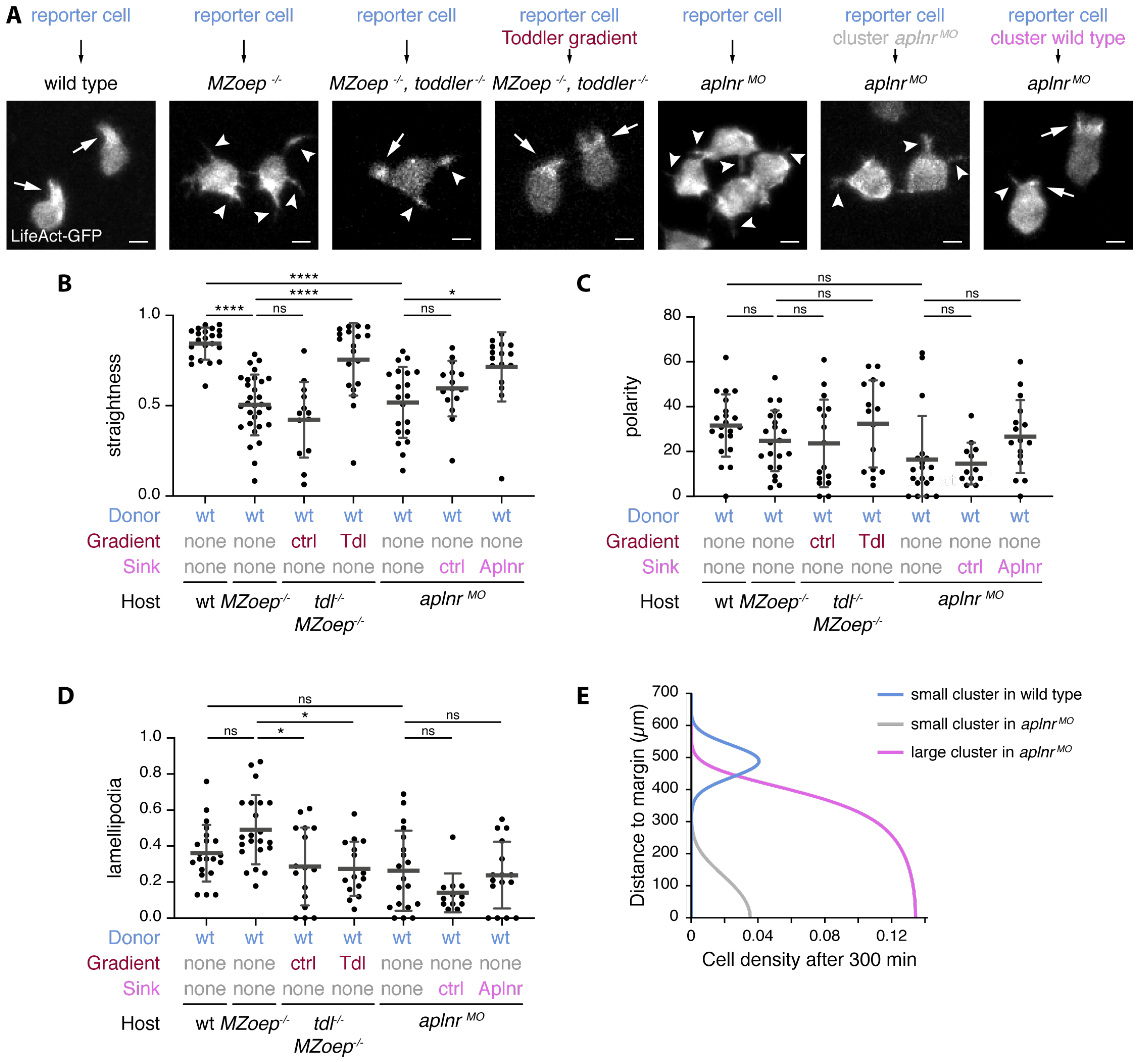

fig. S5. Sink activity of Aplnr-expressing mesodermal cells is required to form a Toddler gradient. (A) Representative confocal images of time-lapse series of transplanted reporter cells in the presence or absence of a mesodermal sink or Toddler gradient, as depicted in **Fig. 4A**. Arrows and arrowheads indicate lamellipodia and filopodia, respectively. **(B)** Quantification of straightness of tracks presented in **Fig. 4B**. **(C)** Quantification of cell polarity of reporter cells from **Fig. 4A-C** represented as percentage of frames in which a cell was polarized. **(D)** Quantification of lamellipodia detected in reporter cells from **Fig. 4A-C** per frame. **(E)** Numerical simulation of scenarios presented in **Fig. 4A** with the same model parameters as in **Figure 3**. Migration of small or large clusters of Aplnr-expressing mesodermal reporter cells, which are able to take up Toddler, was simulated in the presence (wild type) or absence (*aplnr*^{MO}) of Aplnr in host embryos (all other parameters being identical).

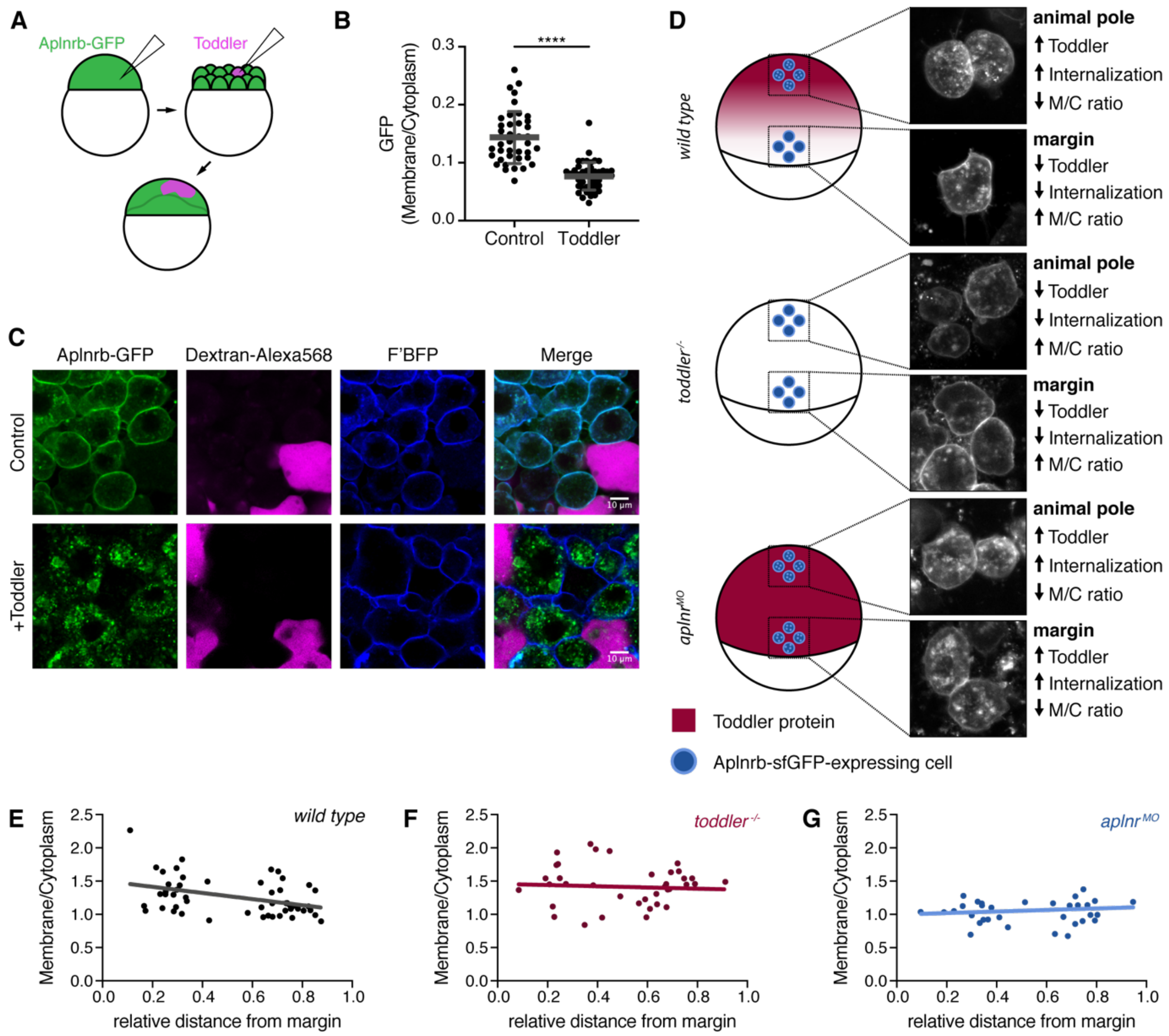

fig. S6. Toddler-induced Aplnrb-sfGFP internalization as a read-out for the Toddler concentration gradient. (A) Schematic representation of experimental set-up. *Aplnrb-GFP* mRNA (green) was injected together with *farnesylated-BFP* mRNA into 1-cell stage embryos to be ubiquitously expressed. Dextran-AlexaFluor568 alone or together with *toddler* mRNA was injected into one blastomere of a 32-cell stage embryo to achieve mosaic expression. **(B)** Quantification of the subcellular localization of Aplnrb-GFP. Data are means ± SD. Significance was determined using unpaired t test; ****, $p < 0.0001$. **(C)** Representative confocal images of Aplnrb-GFP localization in the absence of Toddler (top, n = 32 cells) or under Toddler overexpression conditions (bottom, n = 27 cells). **(D)** Assessment of Aplnrb-sfGFP internalization levels in *toddler*$^{-/-}$ cells transplanted to the animal pole and margin of wild-type (top), *toddler*$^{-/-}$ (middle) and *aplnr*^{MO} (bottom) host embryos. (Left) Schematic representation of transplantation set up and expected Toddler gradient (red) and Aplnrb-sfGFP internalization levels (blue) based on host genotype and position. (Right) Representative images of Aplnrb-sfGFP-expressing *toddler*$^{-/-}$ cells at animal pole and margin in respective host embryos. **(E-G)** Ratio of membrane to cytoplasm fluorescence intensity of transplanted cells relative to their distance to the margin for wild-type (E), *toddler*$^{-/-}$ (F) and *aplnr*^{MO} (G) embryos.

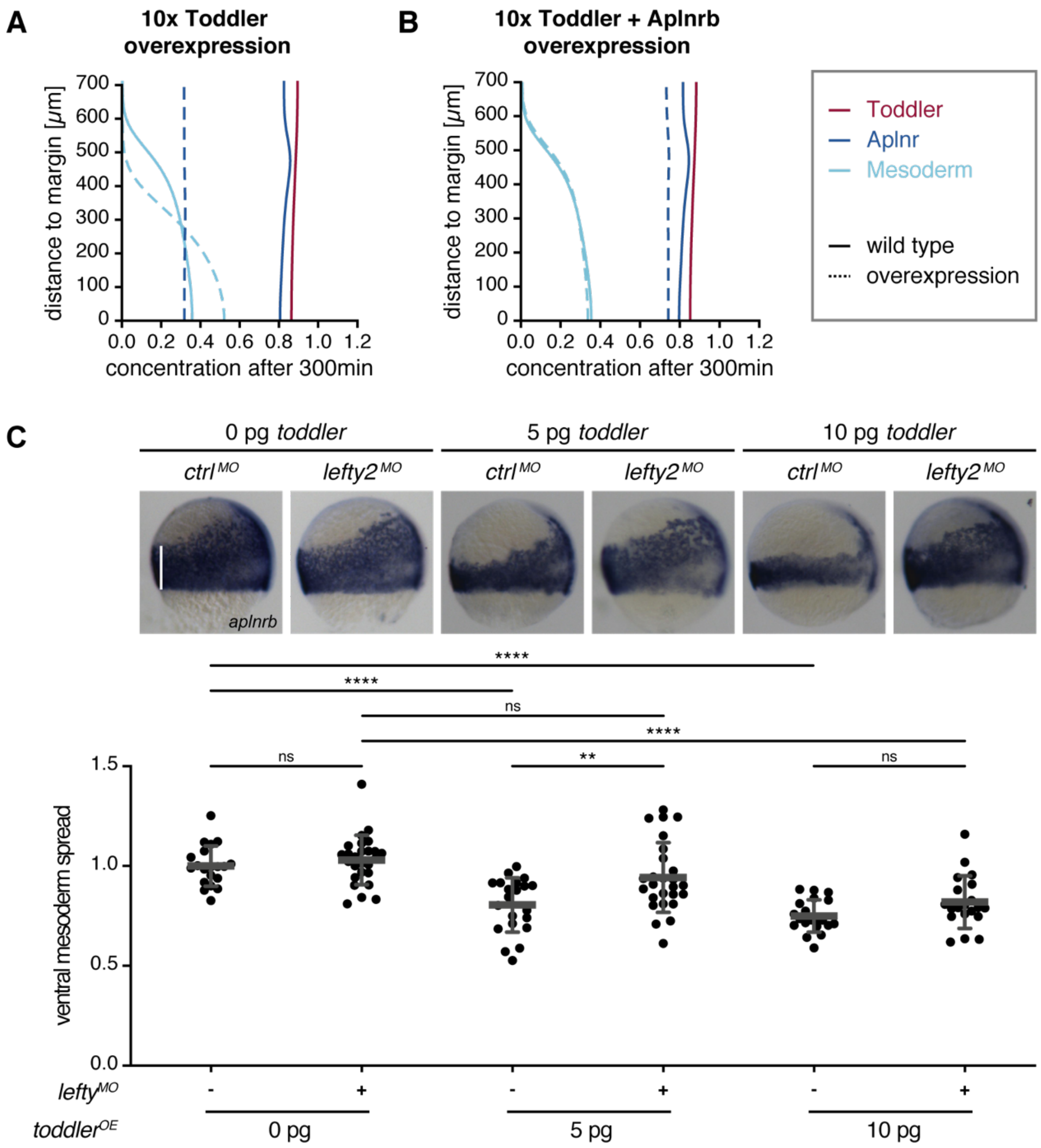

fig. S7. Overexpression of Toddler disrupts self-generated gradient but can be compensated by increasing the number of mesodermal cells. (A) Numerical simulation of mesoderm (light blue) migration upon Toddler (red) overexpression (10-fold, i.e. $T_0 = 10$), assuming a finite capacity of Aplnr (dark blue) to remove Toddler and cells sensing relative Toddler gradients (see **Materials and Methods** for details). Toddler over-expression causes a decrease in Aplnr concentration, which prevents efficient Toddler gradient formation, resulting in slower mesoderm migration. Unbroken lines represent wild-type scenario, dotted lines depict the changes upon Toddler overexpression. **(B)** Numerical simulation of mesoderm migration upon Toddler and Aplnr overexpression (same parameters as in (A) for Toddler; Aplnr overexpression by 2.25-fold, see **Materials and Methods** for details), which rescues normal mesoderm migration. Representation as described in (A). **(C)** Experimental confirmation of the simulation presented in (A-B). Toddler was overexpressed at different concentrations in wild-type embryos (rescuing concentration of *toddler* mRNA injection into 1-cell stage *toddler*$^{-/-}$ embryos is 2 pg) injected with control or *lefty2* MO to assess the compensation of increased Toddler levels upon increase of mesodermal cells (reducing the levels of the Nodal inhibitor Lefty2 increases the amount of Aplnr-expressing mesodermal cells). (Top) Representative *in situ* hybridization images using *aplnrb* as a probe to detect mesodermal cells. Embryos are shown as lateral views, with dorsal on the right. The white vertical line was used to measure the mesoderm spread from the margin towards the animal pole. (Bottom) Quantification of the mesoderm spread in each condition relative to the average mesoderm spread in wild-type embryos. Data are means ± SD. Significance was determined using one-way ANOVA with multiple comparison; ****, $p < 0.0001$; **, $p < 0.01$; n.s., not significant.

Supplementary movie legends

Movie S1 | Internalized wild-type cells polarize and extend actin-rich lamellipodia towards the animal pole. Light sheet time-lapse imaging (time interval: 1 min) of LifeAct-GFP-labelled wild-type cells transplanted to the margin of a wild-type host embryo. Establishment of a polymerized actin network and lamellipodia is marked by accumulation of LifeAct-GFP at the front of the cell. The movie starts after cells have successfully internalized, confirming that cells were of either mesodermal or endodermal cell fate, and shows efficient animal-pole directed migration of these cells. Each frame is a maximum intensity projection of a z-stack. Animal pole is to the top. Scale bar, 10 μm.

Movie S2 | Internalized *toddler*$^{-/-}$ cells fail to polarize and fail to form actin-rich lamellipodia. Light sheet time-lapse imaging (time interval: 1 min) of LifeAct-GFP-labelled *toddler*$^{-/-}$ cells transplanted to the margin of a *toddler*$^{-/-}$ host embryo. Cells display a loss of polarization and lamellipodia formation, as well as the formation of ectopic filopodia around the cell periphery. The movie starts after cells have successfully internalized, confirming that cells were of either mesodermal or endodermal cell fate. Cells fail to move towards the animal pole. Each frame is a maximum intensity projection of a z-stack. Animal pole is to the top. Scale bar, 10 μm.

Movie S3 | A subset of internalized *toddler*$^{-/-}$ cells displays an increased blebbing phenotype. Light sheet time-lapse imaging (time interval: 1 min) of LifeAct-GFP-labelled *toddler*$^{-/-}$ cells transplanted to the margin of a *toddler*$^{-/-}$ host embryo, revealing the lack of actin-rich protrusion. Instead, actin-deficient blebs are formed as observed in a subset of analyzed *toddler*$^{-/-}$ cells. The movie starts after cells have successfully internalized, confirming that cells were of either mesodermal or endodermal cell fate. Cells fail to move towards the animal pole. Each frame is a maximum intensity projection of a z-stack. Animal pole is to the top. Scale bar, 10 μm.

Movie S4 | Confocal imaging of internalized wild-type cells. Confocal time-lapse imaging (time interval: 5 min) of LifeAct-GFP-labelled wild-type cells transplanted to the margin of a wild-type host embryo, confirming morphology and animal pole-directed migration observed in movie S1. Each frame is a maximum intensity projection of a z-stack. Animal pole is to the top. Scale bar, 50 μm.

Movie S5 | Confocal imaging of internalized *toddler*$^{-/-}$ cells. Confocal time-lapse imaging (time interval: 5 min intervals) of LifeAct-GFP-labelled *toddler*$^{-/-}$ cells transplanted to the margin of a *toddler*$^{-/-}$ host embryo, confirming morphology and lack of animal pole-directed migration observed in movie S2. Each frame is a maximum projection of a z-stack. Animal pole is to the top. Scale bar, 50 μm.

Movie S6 | *toddler*$^{-/-}$ cells display normal morphology and migration to the animal pole when transplanted into a wild-type host embryo. Confocal time-lapse imaging (time interval: 5 min) of LifeAct-GFP-labelled *toddler*$^{-/-}$ cells transplanted to the margin of a wild-type host embryo. Each frame is a maximum projection of a z-stack. Animal pole is located towards the top. Scale bar, 50 μm.

Movie S7 | Wild-type cells display defects in protrusion formation, polarization and migration to the animal pole when transplanted into a *toddler*$^{-/-}$ host embryo. Confocal time-lapse imaging (time interval: 5 min interval) of LifeAct-GFP-labelled wild-type cells transplanted to the margin of a *toddler*$^{-/-}$ host embryo. Each frame is a maximum projection of a z-stack. Animal pole is located towards the top. Scale bar, 50 μm.

Movie S8 | Aplnr-expressing cells are attracted by a localized source of Toddler. Confocal time-lapse imaging (time interval: 5 min) of Aplnrb-sfGFP-expressing cells reacting to an ectopic Toddler source. Left: Aplnrb-expressing cells (blue) are placed next to a control source (grey). Middle: Aplnr-expressing cells (blue) are placed next to a Toddler-overexpressing source (red). Right: Aplnr-deficient cells (grey) are placed next to a Toddler-overexpressing source (red). Mesodermal and source cells are labelled with LifeAct-GFP and Dextran-AlexaFluore568, respectively. Each frame is a maximum projection of a z-stack. Source is located towards the top. Scale bar, 20 μm.

Movie S9 | Wild-type reporter cells lose directional migration and polarity in *MZoep* $^{-/-}$ host embryos. Confocal time-lapse imaging (time interval: 5 min interval) of wild-type reporter cells (blue) transplanted into *MZoep* $^{-/-}$ embryos to test for the necessity of a mesendodermal Toddler sink. Left: Reporter cells transplanted to the margin of an *MZoep* $^{-/-}$ host embryo, which is deficient of mesendodermal progenitor cells. Middle: Reporter cells transplanted to the margin of an *MZoep* $^{-/-}$, *toddler* $^{-/-}$ double mutant host embryo, which is deficient of mesendodermal progenitor cells and Toddler expression. A control source (grey) was transplanted to the animal pole. Right: Reporter cells transplanted to the margin of an *MZoep* $^{-/-}$, *toddler* $^{-/-}$ double mutant host embryo, which is deficient of mesendodermal progenitor cells and Toddler expression. A Toddler source (red) was transplanted to animal pole to mimic an ectopic Toddler gradient. Segregation of two cell groups, one clearly moving towards the Toddler source and the other one lagging behind, likely stems from the fact that transplanted cells are a mixture of mesodermal (Aplnrb-expressing and Toddler-responsive) and endodermal (Aplnrb-deficient and non-responsive to Toddler) progenitor cells.. Each frame is a maximum projection of a z-stack. Animal pole is located towards the top. Scale bar, 50 µm.

Movie S10 | Wild-type reporter cells lose directional migration and polarity in *MZoep* $^{-/-}$ host embryos. Confocal time-lapse imaging (time interval: 5 min interval) of wild-type reporter cells (blue) transplanted into *aplnr* ^{MO} embryos to test for the effect of individual versus collective cell migration in an environment of ubiquitous Toddler levels. Left: Reporter cells transplanted to the margin of an *aplnr* ^{MO} host embryo, which forms mesoderm but is deficient in Aplnr expression. Middle: Co-transplantation of reporter cells and a control cluster of Aplnrb-deficient cells (grey) to the margin of an *aplnr* ^{MO} host embryo. Right: Co-transplantation of reporter cells and a cluster of Aplnrb-expressing cells (magenta) to the margin of an *aplnr* ^{MO} host embryo to mimic sink activity. Each frame is a maximum projection of a z-stack. Animal pole is located towards the top. Scale bar, 50 µm.

Table 1 | List of plasmids

Plasmid	mRNA	Restriction/Polymerase	Source
AP242	toddler	BglII/SP6	Pauli et al., 2014 (*10*)
R013	oep	NotI/T7	Zhang et al., 1998 (*60*)
AP552	aplnrb	EcoRV/T7	Pauli et al., 2014 (*10*)
AP606	aplnrb-sfgfp	BglII/SP6	Pauli et al., 2014 (*10*)
R160	lifeact-gfp	XbaI/T3	Raz Lab
R203	human h2b-bfp	EcoRV/SP6	Tsai et al., 2020 (*61*)
R009	human h2b-rfp	NotI/SP6	https://www.addgene.org/53745/
R202	f'bfp	EcoRV/SP6	Shindo et al., 2018 (*62*)

Table 2 | List of primers

Primer name	Sequence
toddler_gt_F	CGACAGAATTTATCGTCTGAGGAAC
toddler_gt_R	TGAAAGTTACATTGGGTTAGAAAGC
oep_gt_F	AGGCCCTCGAGATAAATAACA
oep_gt_R	ACAGCAAACATCAAGAACCTG

Table 3 | Settings for light sheet microscopy

	**Cell morphology**	**Cell tracking**
Magnification/Objective	20x water immersion	10x water immersion
Laser	488 (6%) and 561 (5%)	499 (5%) and 561 (5%)
Exposure time	30 ms	30 ms
Illumination	Dual sided illumination with online fusion	Dual sided illumination with online fusion
Image size	439.1x439.1 µm; 1920x1920 px	772.85x772.85 µm; 1200x1200 px
Z-stack	50 to 80 slices (depending on embryo), 2 µm interval	150 slices, 1.774 µm interval
Time series	500 time points, 30 sec intervals	700 time points, 42 sec intervals

Table 4 | Settings for confocal microscopy

	**Transplantation experiments**	**Aplnrb-GFP internalization**
Maginfication/Objective	10x (air)	20x (air)
Laser	405, 488 and 561	405, 488 and 561
Pinhole	4 AU (120 µm)	1 AU (32 µm)
Averaging	2x	8x
Image size	14.19x14.09 mm; 20467x20322 px	319.45x319.45 µm; 1024x1024 px
Z-stack	9 slices (120 µm)	21 slices (20 µm)
Time series	72 time points (25 for chemokine assays), 5 min interval	Not applicable

Computational modeling

Here, we provide additional details for the modelling of self-generated Toddler gradients during zebrafish gastrulation.

Position of the problem

We write the conservation equation for the concentration of mesoderm cells $m(x,t)$ and secreted Toddler $T(x,t)$ as a function of time t and position x along the animal-vegetal axis (where $x = 0$ is the position of the margin, and where we restrict ourselves to a one-dimensional description thanks to the radial symmetry of the problem):

$$\begin{cases} \partial_t m = D_m \partial_{xx} m - \partial_x m v \\ \partial_t T = D_T \partial_{xx} T + \dfrac{T_0(x) - T - \alpha m T}{\tau_T} \end{cases}$$

In this description, mesodermal cell concentration can change from i) free diffusion and ii) directional motion at speed v, while Toddler concentration can change from i) free diffusion, ii) production (from ectoderm cells), iii) intrinsic degradation and iv) mesoderm consumption. We have denoted D_m and D_T as the diffusion coefficient of mesoderm cells (in the absence of directed motion) and Toddler molecules, respectively, $T_0(x)$ as the target concentration of Toddler (which can be spatially modulated) in the absence of any mesoderm consuming it, τ_T as the timescale of intrinsic Toddler degradation and α as the consumption rate of Toddler from mesoderm cells (larger density of mesoderm cells signifying more receptor density for Toddler degradation – note that this implicitly assumes that receptor density per cell is constant, an assumption that we relax below). For this equation we must additionally specify a dependency between directed cell migration velocity v and Toddler concentration. How cells sense gradients is an area of active study, and different non-linear as well as adaptative responses have been uncovered in particular while interpreting GPCR signalling gradients (see for instance review by Jin (*56*)). Here, we explored two simple limits of gradient sensing: $v = \beta \partial_x T$ (i.e. cells move up an *absolute* gradient of Toddler) or $v = \beta \frac{\partial_x T}{T}$ (i.e. cells move up a *relative* gradient in Toddler) - with β denoting in each case the strength of the coupling. We also note that this equation makes the important approximation (which we will come back to below in **fig. S3C**) that Toddler gradients only impacts the average "advective" velocity of cells v and not their random motility coefficient D_m. This is a coarse-grained description which can be made because the cell velocity in response to a Toddler gradient does not necessarily require a Toddler-dependent change of the instantaneous cell speed, but can arise from a partial bias in their random walk that is caused by a more persistent directionality in cell polarity triggered by the local Toddler gradient. Similarly, in our 1D model the velocity v (in the x direction) is proportional to the gradient of Toddler. In a 2D model, this proportional relationship could have different origins, as both the absolute velocity V and the polarity angle θ need to be considerd, while in our case only the projection along the x direction $v = V\, sin\theta$ matters for the problem at hand. For instance, the gradient could either act directly on the absolute velocity V or only on reorienting the polarity angle θ (*57*) although both result in changes in the 1D velocity v.

Finally, we specify boundary and initial conditions for this problem at the margin: the Toddler protein and mesodermal cells cannot escape at the margin, leading to no-flux boundary conditions $\partial_x T(x=0)=0$ and $\partial_x m(x=0)=0$. While the initial conditions for Toddler are largely irrelevant and given its continuous production simplest defined as $T(x,0)=T_0$, they are key for mesoderm specification. We therefore first assume that the mesodermal cell number is fixed and initially concentrated very close to the margin: $m(x,0)=M_0\delta(x,0)$.

Key length and timescales in the problem

From these equations, two natural scales emerge: a time scale τ_T, which represents the timescale of Toddler turnover, and a length scale $L=\sqrt{D_T\tau_T}$, which represents the distance at which Toddler produced by a localized source is degraded. This is, for instance, important during our rescue experiments, during which we place Toddler-expressing cells at the animal pole (**Fig. 4A**, forth from the right): Toddler is predicted to decay exponentially from the source cell location, at a length scale of L.

Parameter constraints, fitting and non-dimensionalization

A number of parameters can be constrained in this model. Toddler diffusion in particular can be calculated from the size of the Toddler molecule (around 4 kDa) from the Stokes-Einstein relationship. For instance, Lefty, Cyclops or Squint, which have around 10 times the molecular weight of Toddler (and thus are expected to have 2-3 times the hydrodynamical radius) were shown to have free diffusion coefficients in zebrafish embryos of $D_T \approx 20\ \mu m^2.s^{-1}$ (*27*). Therefore, we can estimate that for Toddler $D_T \approx 50\ \mu m^2.s^{-1}$ or $D_T \approx 3.10^3 \mu m^2.min^{-1}$. To estimate the time scale τ_T of Toddler degradation, we have made use of *MZoep* $^{-/-}$, *toddler* $^{-/-}$ double mutant embryos, which are devoid of both endogenous Toddler production and mesoderm-induced Toddler degradation. We injected 1 µg of Toddler peptide and performed a time course analysis of Toddler degradation (**fig. S4F-G**). This showed a roughly exponential decay, as predicted by our linear model, and from which we could extract $\tau_T \approx 120$ min (**fig. S4F-G**). We also compared this exponential to a linear fit $1-t/t_T$, and used Akaike Information Criterion to see whether this was a more likely model. However, the comparison favored the exponential fit (Difference in AICc of 1.475). Interestingly, this value is in the same order of magnitude as Lefty, Cyclops or Squint during early zebrafish embryo morphogenesis (*27*). Together, this predicts a length scale for Toddler propagation of $L=\sqrt{D_T\tau_T} \approx 600\ \mu m$, which, interestingly, is of comparable scale to the embryo itself. Note that modelling the full complexity of diffusion in a cell-fluid mixture would give rise to slightly different prefactors (up to a factor 2) (*58*), though, consistent with previous work on self-generated gradients, this would have little quantitative impact on the global dynamics of mesendoderm migration, as a change in diffusion by orders of magnitude would be required to give qualitatively different dynamics (*59*). Taken together, this length scale is consistent with the ability of marginal cells to sense Toddler-expressing cells far away at the animal pole, and to restore animal pole-directed migration as demonstrated in the experiments of **Fig. 2F and 4**.

Next, we examined the movements of *toddler* $^{-/-}$ cells transplanted into a *toddler* $^{-/-}$ background. As these cells show no measurable directed motion ($v=0$), we reasoned we could use these experiments

to constrain the value of free cell diffusion D_m. We found that these cells diffused on a length scale of approximately $25\ \mu m$ from the margin during the 30 min of the timescale (**fig. S4K-L**), leading us to a rough estimate of $D_m \approx 20\ \mu m^2 . min^{-1}$.

Once we rescale all time scales by τ_T, all length scales by L:

$$\begin{cases} \partial_t m = \dfrac{D_m}{D_T} \partial_{xx} m - \dfrac{\beta}{D_T} \partial_x (m \partial_x T) \\ \partial_t T = \partial_{xx} T + T_0 - T - \alpha m T \end{cases}$$

Note that this neglects advective terms in the Toddler equation (for instance cell/fluid movements transporting Toddler). This is a safe assumption given the order of magnitude difference between the two. Estimating a Peclet number yields $Lv/D_T \approx 0.04$, so that free diffusion is largely dominant. We can further rescale Toddler by its maximal concentration T_0, and mesoderm by its initial total amount M_0 leading to

$$\begin{cases} \partial_t m = \dfrac{D_m}{D_T} \partial_{xx} m - \dfrac{\beta T_0}{D_T} \partial_x (m \partial_x T) \\ \partial_t T = \partial_{xx} T + 1 - T - \alpha M_0 m T \end{cases}$$

Thus, in addition to the length and time scales (which have been independently measured), this shows that the problem now only depends on 3 rescaled parameters: the relative diffusion coefficients of mesoderm and Toddler $\frac{D_m}{D_T}$ (which is strongly constrained by our measurements, and found to be very small), the rescaled consumption of Toddler from mesoderm αM_0, and the rescaled coupling from Toddler gradient to directed mesoderm speeds $\frac{\beta T_0}{D_T}$. These two last parameters are harder to independently measure and should therefore be considered as fitting parameters in the theory.

Importantly, however, analysis of the system of equations above finds that these last two parameters can largely be coarse-grained into a single one, with their product being the most relevant parameter: this is because α controls how strong of a gradient of Toddler is created, and β how strongly this gradient is interpreted, so that high α – low β and low α – high β give rise to similar velocities. Numerical simulations keeping the product $\alpha\beta$ constant, but changing each by several orders of magnitude confirmed this (see **fig. S4C** where we multiply β by 5 and divide α by 5, giving nearly identical results to **fig. S4A**), although this effect would break down at very high α (when mesoderm consumption of Toddler would be so strong that the Toddler concentration reaches values close to zero).

Another approximation of the model that we wished to verify was that the random motility of cells (represented by the diffusion coefficient D_m) was unaffected by the local Toddler gradient. To verify this, we quantitatively analyzed the experiments in which cells with or without Aplnr were transplanted at a distance from Toddler-secreting or Toddler-deficient cells (**Fig. 2A-C**). We quantified the average displacement of cells over 15 min, either in the direction of the source (y-axis) or perpendicular to it (x-axis), and generated probability distributions for each case. As expected from a purely random and diffusive process in the x-direction, all three conditions displayed Gaussian distributions in step size centered around zero average displacement (**fig. S3C**). Importantly, the standard deviation (proportional to D_m) was nearly identical in all three cases, arguing that random motility is unaffected

by either the presence of a Toddler gradient or Apelin receptor expression. Interestingly, when looking at the same distribution in the y-direction (towards the source), we found again that the standard deviation of the displacement was comparable between conditions (and also to its value in the x-direction, as expected from a random walk, **fig. S3C**). The only difference for the Toddler-Apelin receptor pair was that the best-fit Gaussian distribution was not centered around 0, but instead around a non-zero average velocity value of $0.3\ \mu m/min$ – as expected for a biased random walk and our model in which Toddler gradients only act on the advective velocity *v* (**fig. S3C**, see legends for detailed statistics and fitting).

Finally, although the model as defined above assumes that all cells internalize at the same time (initial Delta function at x=0 in the initial condition $m(x,0) = M_0\delta(x,0)$), the experimental situation is more gradual, with numbers of internalizing cells showing a broad temporal peak with typical variance σ of an hour (*13*). This can easily be taken into account by assuming that the initial condition is now zero mesoderm cells $m(x,0) = 0$, but adding a gradual source term in the conservation equation for mesoderm cells: $\partial_t m = \frac{D_m}{D_T}\partial_{xx} m - \frac{\beta T_0}{D_T}\partial_x(m\partial_x T) + I(x,t)$, where $I(x,t)$ is the spatio-temporal dynamics of internalization. Thus, we take in the simulations $I(x,t) = M_0 H(l-x)e^{-\frac{t^2}{2\sigma^2}}$: internalization only occurs close to the margin (represented by a Heaviside function decaying at $l = 50\ \mu m$), and on timescales $\sigma = 1h$ (*13*). Although these are the parameters that we show in **Fig. 3**, we also ran simulations with the previous initial condition (synchronous internalization of all mesendoderm cells) and found very similar results for both wild type and *toddler*$^{-/-}$ simulations (see **fig. S4A-E**).

Thus, in the following, we only fit $\alpha\beta$ in the theory (simulations from main text are made for $\alpha M_0 = 1$). $\alpha\beta$ is essentially proportional to the speed of migrating cells up a self-generated gradient. As we found an average speed of $v \approx 0.08\ \mu m/min$, this means $\alpha\beta \approx 10^4$ in our unit simulations.

With the model fully parametrized in this manner, we turned to its predictions on a number of non-trivial features, such as the spatiotemporal density/velocity profiles of mesoderm migration in wild type and *toddler*$^{-/-}$ mutant, or transplantation assays (**Fig. 3, S5D, S7A,B**).

Model predictions

We first consider the case of *toddler*$^{-/-}$, in which directed cell migration is negligible ($v = 0$). Because the margin constitutes a hard boundary, internalized cells are expected to still migrate upwards to some degree according to a diffusive process with coefficient D_m. Given our estimate of D_m from short-term trajectories, we thus asked how much cells were predicted to travel by pure diffusion (1D along the animal-vegetal axis) in the Δt=3.5 hours between internalization and the 75% epiboly stage. These simulations predicted around 200 µm, compared to 600 µm for wild type (**fig. S4H**). To confirm these predictions experimentally, we measured the intensity profiles of mesoderm markers (*aplnrb*) by *in situ* hybridization assays in *toddler*$^{-/-}$ compared to the wild-type embryos along the animal-vegetal axis as a proxy for the mesoderm concentration (**fig. S4I-J**). We found that the mesoderm concentration profile in *toddler*$^{-/-}$ embryos decayed around twice as fast as in wild-type embryos. It is important to note that uncertainty in the exact diffusion coefficient of mesoderm cells, or the possibility of small,

residual, non-zero directionality in the migration of *toddler*$^{-/-}$ cells could explain the slightly stronger phenotype in the model.

In the presence of self-generated gradients, the system organizes into a travelling-wave solution, as expected from the literature (*23*), where cells adopt a non-zero net polarity/velocity towards the animal pole, as observed experimentally (**Fig. 2B-C,E**). Although this self-organized collective migration is robust to the details of the parameters, such as the effective diffusion length scale L for Toddler, such parameters do have an effect on the detailed spatiotemporal profiles of mesoderm migration. For very local Toddler diffusion (small L), only a few cells at the very edge sense the self-generated gradient, which causes them to initially migrate very fast (see **fig. S4D** for a simulation with $\tau_T = 10\,s$, so that the length scale L is around 20 µm, i.e. the cell size). However, this creates a concentration gradient of mesoderm cells, which in turn causes a concentration gradient of Toddler, which does not stem from diffusion, but rather the differential degradation of Toddler caused by the spatial differences in mesoderm density. Thus, cells can still migrate in a self-generated manner, although the cellular density gradient is more pronounced than the "front-like" solutions shown in **Fig. 3** (limit of large effective Toddler diffusion relevant here as the length scale L is of the order of the embryo size as described above).

Comparing these predictions to our tracking data of mesoderm cells (marked by *drl:GFP*) undergoing migration towards the animal pole after internalization, we found similar qualitative features, with cells at the edge displaying the largest velocity, which decreased both as a function of time and distance from the edge. More quantitatively, we compared kymographs for the cellular velocity as a function of position, which equals the distance from margin and time (**Fig. 3H**). It is important to note that in the kymographs we show the total effective cell velocity as measured by cell tracking, which represents the sum of the advective velocity and the diffusive flux, which can have directional contribution in the presence of a density gradient. The total flux of cells reads as $J_{tot} = m\beta\partial_x T - D_m\partial_x m,$ in which the first term is the advective contribution proportional to the local Toddler gradient, and the second is the diffusive flux. We thus define $v_{tot} = J_{tot}/m = \beta\partial_x T - D_m\partial_x m/m$ as the total average velocity of mesodermal cells at position x, which is plotted in **Fig. 3H**. Importantly, while we predict that the contribution of diffusion is rather small compared to advection (see **fig. S4E** for a simulation with zero mesendoderm free diffusion, $D_m = 0$) in wild type, the latter becomes dominant in *toddler*$^{-/-}$, in which advection is close to zero.

Effect of number of transplanted cells on the resulting dynamics

As discussed in the main text, the mechanism of self-generated gradients that we propose relies on a collective effect, for which the number of cells at the margin matters as it dictates the strength of gradient-shaping. To further explore the effect of sink function and cell number, we refined the model to include different cell types: regular Aplnr-expressing mesoderm cells $m(x,t)$, which degrade Toddler, and Aplnr-deficient cells which do not degrade Toddler $m_0(x,t)$. The equation on $m(x,t)$ is exactly the same as before, as is the equation on Toddler (only $m(x,t)$ participate in Toddler degradation, while $m_0(x,t)$ does not enter the Toddler equation). The equation on $m_0(x,t)$ reads

$\partial_t m_0 = \frac{D_m}{D_T} \partial_{xx} m_0$ (i.e. no directed migration term). As we show in **fig. S5D**, simulating transplants of small numbers of wild-type cells in *aplnr*^{MO} embryos (large m_0 density, low m density) resulted in little migration, while transplants of small numbers of wild-type cells in wild-type density was effectively the same as regular wild-type migration (as transplanted cells are identical to the surroundings). On the other hand, simulating large clusters of wild-type cells in *aplnr*^{MO} embryos (large m_0 density, intermediate m density) resulted in an intermediary phenotype (**fig. S5D**), as seen in the data (**Fig. 4**).

Overexpression of Toddler and Apelin receptor

We next consider the effect of overexpression of Toddler, which has shown to give rise to defects in upward migration (*10*). This is modelled by a change in the baseline production of Toddler T_0:

$$\partial_t T = \partial_{xx} T + T_0 - T - \alpha M_0 m T$$

where previously we had non-dimensionalized the problem to $T_0 = 1$. When considering different T_0, the assumption of absolute vs. relative gradient sensing (resp. $v = \beta \partial_x T$ or $v = \beta \frac{\partial_x T}{T}$) does impact the resulting prediction: for absolute gradient sensing, the gradient (and thus migration) increases with T_0, whereas it is almost insensitive to T_0 for relative gradient sensing. However, it should be noted that the equation above implicitly assumes that mesoderm cells (via their Apelin receptors) can take up arbitrary amounts of Toddler ligand. Although this may be correct for the wild-type condition, this situation might not generally hold true for overexpression phenotypes, especially as Apelin receptors are internalized with Toddler, which might result in not enough Apelin receptor left on membranes to sense and uptake Toddler. To take this latter feature of GPCR signalling into account, we supplemented the equations above with a conservation equation for the concentration of Apelin receptors within a cell $r(x,t)$:

$$\tau_r \partial_t r + \partial_x (r v) = r_0 - r - \alpha M_0 r T$$

Note that there are no diffusion terms in this equation, as Apelin receptors don't spatially exchange between cells, although they are "transported" spatially together with the movements of mesoderm cells (advective term putting r in the co-moving frame of cells). This first-order equation assumes that there is a baseline equilibrium of Apelin receptor at the membrane (time scale of recycling τ_r, equilibrium concentration r_0), but that each event of Toddler internalization also removes an Apelin receptor. This is the same sink term as in the Toddler equation, now rewritten to depend also on r :

$$\partial_t T = \partial_{xx} T + T_0 - T - \alpha M_0 T r m$$

Note that the latter term also is multiplied by the mesendoderm concentration m, as we have defined $r(x,t)$ as the single-cell concentration of Apelin receptors.

When simulating these equations with $r_0 = 1$ and $\tau_r = 30$ min (although these values had little bearing on the results), we found very similar dynamics as in previous simulations, with Apelin receptor concentration very slightly increased at the back of the mesoderm edge (where Toddler concentration is lower). However, when simulating a 10x increase in Toddler production ($T_0 = 10$), which was close to concentrations previously reported to induce a Toddler overexpression phenotype (*10*) **(fig. S7C),** we found that this now resulted in an impaired migration (**fig. S7A**), which was due to a much lower concentration of r (due to receptor saturation through Toddler binding), and therefore an impairment

of gradient formation. The more the Toddler production was increased, the stronger the defect in migration was. However, increasing the production of Apelin receptor r_0 could restore the normal wild-type phenotype (**fig. S7B**). The exact amount of Aplnr to fully compensate for the 10x Toddler overexpression depends on the turnover rate of Apelin receptors; for $\tau_r = 30$ min, $r_0 = 2.25$ was required to restore the normal migration profile from $T_0 = 10$. Interestingly, this closely matches previous experimental observations of an epistatic relationship between Toddler and Apelin receptors (**fig. S7C**).

REFERENCES AND NOTES

1. L. Solnica-Krezel, D. S. Sepich, Gastrulation: Making and shaping germ layers. *Annu. Rev. Cell Dev. Biol.* **28**, 687–717 (2012).

2. L. Solnica-Krezel, Conserved patterns of cell movements during vertebrate gastrulation. *Curr. Biol.* **15**, R213–R228 (2005).

3. L. A. Rohde, C. P. Heisenberg, Zebrafish gastrulation: Cell movements, signals, and mechanisms. *Int. Rev. Cyt.* **261**, 159–192 (2007).

4. D. Pinheiro, C. P. Heisenberg, in *Current Topics in Developmental Biology* (Academic Press Inc., 2020), vol. 136, pp. 343–375.

5. B. Feldman, M. A. Gates, E. S. Egan, S. T. Dougan, G. Rennebeck, H. I. Sirotkin, A. F. Schier, W. S. Talbot, Zebrafish organizer development and germ-layer formation require nodal-related signals. *Nature* **395**, 181–185 (1998).

6. A. F. Schier, Nodal morphogens. *Cold Spring Harb. Perspect. Biol.* **1**, a003459 (2009).

7. A. F. Schier, W. S. Talbot, Molecular genetics of axis formation in zebrafish. *Annu. Rev. Genet.* **39**, 561–613 (2005).

8. M. L. Williams, L. Solnica-Krezel, Regulation of gastrulation movements by emergent cell and tissue interactions. *Curr. Opin. Cell Biol.* **48**, 33–39 (2017).

9. C. B. Kimmel, W. W. Ballard, S. R. Kimmel, B. Ullmann, T. F. Schilling, Stages of embryonic development of the zebrafish. *Dev. Dyn.* **203**, 253–310 (1995).

10. A. Pauli, M. L. Norris, E. Valen, G. L. Chew, J. A. Gagnon, S. Zimmerman, A. Mitchell, J. Ma, J. Dubrulle, D. Reyon, S. Q. Tsai, J. K. Joung, A. Saghatelian, A. F. Schier, Toddler: An embryonic signal that promotes cell movement via apelin receptors. *Science* **343**, 1248636 (2014).

11. S. C. Chng, L. Ho, J. Tian, B. Reversade, ELABELA: A hormone essential for heart development signals via the apelin receptor. *Dev. Cell* **27**, 672–680 (2013).

12. M. L. Norris, A. Pauli, J. A. Gagnon, N. D. Lord, K. W. Rogers, C. Mosimann, L. I. Zon, A. F. Schier, Toddler signaling regulates mesodermal cell migration downstream of Nodal signaling. *eLife* **6**, e22626 (2017).

13. P. J. Keller, A. D. Schmidt, J. Wittbrodt, E. H. K. Stelzer, Reconstruction of zebrafish early embryonic development by scanned light sheet microscopy. *Science* **322**, 1065–1069 (2008).

14. R. H. Row, J. L. Maître, B. L. Martin, P. Stockinger, C. P. Heisenberg, D. Kimelman, Completion of the epithelial to mesenchymal transition in zebrafish mesoderm requires Spadetail. *Dev. Biol.* **354**, 102–110 (2011).

15. Y. Belotti, D. McGloin, C. J. Weijer, Effects of spatial confinement on migratory properties of *Dictyostelium discoideum* cells. *Commun. Integr. Biol.* **14**, 5–14 (2021).

16. M. Bergert, S. D. Chandradoss, R. A. Desai, E. Paluch, Cell mechanics control rapid transitions between blebs and lamellipodia during migration. *Proc. Natl. Acad. Sci. U.S.A.* **109**, 14434–14439 (2012).

17. V. Venturini, F. Pezzano, F. C. Castro, H. M. Häkkinen, S. Jiménez-Delgado, M. Colomer-Rosell, M. Marro, Q. Tolosa-Ramon, S. Paz-López, M. A. Valverde, J. Weghuber, P. Loza-Alvarez, M. Krieg, S. Wieser, V. Ruprecht, The nucleus measures shape changes for cellular proprioception to control dynamic cell behavior. *Science* **370**, eaba2644 (2020).

18. C. S. Helker, A. Schuermann, C. Pollmann, S. C. Chng, F. Kiefer, B. Reversade, W. Herzog, The hormonal peptide Elabela guides angioblasts to the midline during vasculogenesis. *eLife* **4**, e06726 (2015).

19. H. Blaser, S. Eisenbeiss, M. Neumann, M. Reichman-Fried, B. Thisse, C. Thisse, E. Raz, Transition from non-motile behaviour to directed migration during early PGC development in zebrafish. *J. Cell Sci.* **118**, 4027–4038 (2005).

20. G. Venkiteswaran, S. W. Lewellis, J. Wang, E. Reynolds, C. Nicholson, H. Knaut, Generation and dynamics of an endogenous, self-generated signaling gradient across a migrating tissue. *Cell* **155**, 674–687 (2013).

21. E. Donà, J. D. Barry, G. Valentin, C. Quirin, A. Khmelinskii, A. Kunze, S. Durdu, L. R. Newton, A. Fernandez-Minan, W. Huber, M. Knop, D. Gilmour, Directional tissue migration through a self-generated chemokine gradient. *Nature* **503**, 285–289 (2013).

22. L. Tweedy, D. A. Knecht, G. M. Mackay, R. H. Insall, Self-generated chemoattractant gradients: Attractant depletion extends the range and robustness of chemotaxis. *PLoS Biol.* **14**, e1002404 (2016).

23. L. Tweedy, O. Susanto, R. H. Insall, Self-generated chemotactic gradients—Cells steering themselves. *Curr. Opin. Cell Biol.* **42**, 46–51 (2016).

24. J. A. Farrell, Y. Wang, S. J. Riesenfeld, K. Shekhar, A. Regev, A. F. Schier, Single-cell reconstruction of developmental trajectories during zebrafish embryogenesis. *Science* **360**, eaar3131 (2018).

25. A. F. Schier, S. C. Neuhauss, K. A. Helde, W. S. Talbot, W. Driever, The one-eyed pinhead gene functions in mesoderm and endoderm formation in zebrafish and interacts with no tail. *Development* **124**, 327–342 (1997).

26. K. Gritsman, J. Zhang, S. Cheng, E. Heckscher, W. S. Talbot, A. F. Schier, The EGF-CFC protein one-eyed pinhead is essential for nodal signaling. *Cell* **97**, 121–132 (1999).

27. P. Müller, K. W. Rogers, B. M. Jordan, J. S. Lee, D. Robson, S. Ramanathan, A. F. Schier, Differential diffusivity of nodal and lefty underlies a reaction-diffusion patterning system. *Science* **336**, 721–724 (2012).

28. C. Mosimann, D. Panáková, A. A. Werdich, G. Musso, A. Burger, K. L. Lawson, L. A. Carr, K. R. Nevis, M. K. Sabeh, Y. Zhou, A. J. Davidson, A. Dibiase, C. E. Burns, C. G. Burns, C. A. Macrae, L. I. Zon, Chamber identity programs drive early functional partitioning of the heart. *Nat. Commun.* **6**, 1–10 (2015).

29. P. Devreotes, A. R. Horwitz, Signaling networks that regulate cell migration. *Cold Spring Harb. Perspect. Biol.* **7**, a005959 (2015).

30. N. J. Pavlos, P. A. Friedman, GPCR signaling and trafficking: The long and short of it. *Trends Endocrinol. Metab.* **28**, 213–226 (2017).

31. A. L. van Boxtel, J. E. Chesebro, C. Heliot, M. C. Ramel, R. K. Stone, C. S. Hill, A temporal window for signal activation dictates the dimensions of a nodal signaling domain. *Dev. Cell* **35**, 175–185 (2015).

32. L. Tweedy, R. H. Insall, Self-generated gradients yield exceptionally robust steering cues. *Front. Cell Dev. Biol.* **8**, 133 (2020).

33. A. Shellard, R. Mayor, Collective durotaxis along a self-generated stiffness gradient in vivo. *Nature* **600**, 690–694 (2021).

34. J. M. Haugh, Deterministic model of dermal wound invasion incorporating receptor-mediated signal transduction and spatial gradient sensing. *Biophys. J.* **90**, 2297–2308 (2006).

35. S. J. Streichan, G. Valentin, D. Gilmour, L. Hufnagel, Collective cell migration guided by dynamically maintained gradients. *Phys. Biol.* **8**, 045004 (2011).

36. C. Scherber, A. J. Aranyosi, B. Kulemann, S. P. Thayer, M. Toner, O. Iliopoulos, D. Irimia, Epithelial cell guidance by self-generated EGF gradients. *Integr. Biol. (Camb).* **4**, 259–269 (2012).

37. J. Alanko, M. C. Ucar, N. Canigova, J. Stopp, J. Schwarz, J. Merrin, E. Hannezo, M. Sixt, Sinking the way: A dual role for CCR7 in collective leukocyte migration. bioRxiv 2022.02.22.481445 (2022).

38. M. Wong, L. R. Newton, J. Hartmann, M. L. Hennrich, M. Wachsmuth, P. Ronchi, A. Guzmán-Herrera, Y. Schwab, A. C. Gavin, D. Gilmour, Dynamic buffering of extracellular chemokine by a dedicated scavenger pathway enables robust adaptation during directed tissue migration. *Dev. Cell* **52**, 492–508.e10 (2020).

39. C. Carmona-Fontaine, H. K. Matthews, S. Kuriyama, M. Moreno, G. A. Dunn, M. Parsons, C. D. Stern, R. Mayor, Contact inhibition of locomotion in vivo controls neural crest directional migration. *Nature* **456**, 957–961 (2008).

40. A. Roycroft, R. Mayor, Molecular basis of contact inhibition of locomotion. *Cell. Mol. Life Sci.* **73**, 1119–1130 (2016).

41. E. Hannezo, C. P. Heisenberg, Mechanochemical feedback loops in development and disease. *Cell* **178**, 12–25 (2019).

42. C. P. Heisenberg, Y. Bellaïche, Forces in tissue morphogenesis and patterning. *Cell* **153**, 948–962 (2013).

43. L. Freyer, C. W. Hsu, S. Nowotschin, A. Pauli, J. Ishida, K. Kuba, A. Fukamizu, A. F. Schier, P. A. Hoodless, M. E. Dickinson, A. K. Hadjantonakis, Loss of apela peptide in mice causes low penetrance embryonic lethality and defects in early mesodermal derivatives. *Cell Rep.* **20**, 2116–2130 (2017).

44. M. Smutny, Z. Ákos, S. Grigolon, S. Shamipour, V. Ruprecht, D. Čapek, M. Behrndt, E. Papusheva, M. Tada, B. Hof, T. Vicsek, G. Salbreux, C. P. Heisenberg, Friction forces position the neural anlage. *Nat. Cell Biol.* **19**, 306–317 (2017).

45. X. X. I. Zeng, T. P. Wilm, D. S. Sepich, L. Solnica-Krezel, Apelin and its receptor control heart field formation during zebrafish gastrulation. *Dev. Cell* **12**, 391–402 (2007).

46. I. C. Scott, B. Masri, L. A. D'Amico, S. W. Jin, B. Jungblut, A. M. Wehman, H. Baier, Y. Audigier, D. Y. R. Stainier, The G protein-coupled receptor Agtrl1b regulates early development of myocardial progenitors. *Dev. Cell* **12**, 403–413 (2007).

47. A. Agathon, B. Thisse, C. Thisse, Morpholino knock-down of Antivin1 and Antivin2 upregulates nodal signaling. *Genesis* **30**, 178–182 (2001).

48. S. Paskaradevan, I. C. Scott, The Aplnr GPCR regulates myocardial progenitor development via a novel cell-non-autonomous, $G\alpha_{i/o}$ protein-independent pathway. *Biol. Open* **1**, 275–285 (2012).

49. K. Jaqaman, D. Loerke, M. Mettlen, H. Kuwata, S. Grinstein, S. L. Schmid, G. Danuser, Robust single-particle tracking in live-cell time-lapse sequences. *Nat. Methods* **5**, 695–702 (2008).

50. R. Jonker, A. Volgenant, A shortest augmenting path algorithm for dense and sparse linear assignment problems. *Comput. Secur.* **38**, 325–340 (1987).

51. F. Pedregosa, G. Varoquaux, A. Gramfort, V. Michel, B. Thirion, O. Grisel, M. Blondel, P. Prettenhofer, R. Weiss, V. Dubourg, J. Vanderplas, A. Passos, D. Cournapeau, M. Brucher, M. Perrot, É. Duchesnay, Scikit-learn: Machine learning in python. *J. Mach. Learn. Res.* **12**, 2825–2830 (2011).

52. S. van der Walt, J. L. Schönberger, J. Nunez-Iglesias, F. Boulogne, J. D. Warner, N. Yager, E. Gouillart, T. Yu; scikit-image contributors, Scikit-image: Image processing in python. *PeerJ.* **2014**, e453 (2014).

53. I. Kasa, A circle fitting procedure and its error analysis. *IEEE Trans. Instrum. Meas.* **IM–25**, 8–14 (1976).

54. C. Thisse, B. Thisse, High-resolution in situ hybridization to whole-mount zebrafish embryos. *Nat. Protoc.* **3**, 59–69 (2008).

55. L. Ho, S. Y. X. Tan, S. Wee, Y. Wu, S. J. C. Tan, N. B. Ramakrishna, S. C. Chng, S. Nama, I. Szczerbinska, Y. S. Chan, S. Avery, N. Tsuneyoshi, H. H. Ng, J. Gunaratne, N. R. Dunn, B. Reversade, ELABELA is an endogenous growth factor that sustains hESC self-renewal via the PI3K/AKT pathway. *Cell Stem Cell* **17**, 435–447 (2015).

56. T. Jin, Gradient sensing during chemotaxis. *Curr. Opin. Cell Biol.* **25**, 532–537 (2013).

57. N. A. Hill, D. P. Häder, A biased random walk model for the trajectories of swimming micro-organisms. *J. Theor. Biol.* **186**, 503–526 (1997).

58. P. Recho, A. Hallou, E. Hannezo, Theory of mechanochemical patterning in biphasic biological tissues. *Proc. Natl. Acad. Sci. U.S.A.* **116**, 5344–5349 (2019).

59. L. Tweedy, P. A. Thomason, P. I. Paschke, K. Martin, L. M. Machesky, M. Zagnoni, R. H. Insall, Seeing around corners: Cells solve mazes and respond at a distance using attractant breakdown. *Science* **369**, eaay9792 (2020).

60. J. Zhang, W. S. Talbot, A. F. Schier, Positional cloning identifies zebrafish one-eyed pinhead as a permissive EGF-related ligand required during gastrulation. *Cell* **92**, 241–251 (1998).

61. T. Y. C. Tsai, M. Sikora, P. Xia, T. Colak-Champollion, H. Knaut, C. P. Heisenberg, S. G. Megason, An adhesion code ensures robust pattern formation during tissue morphogenesis. *Science* **370**, 113–116 (2020).

62. A. Shindo, A. Audrey, M. Takagishi, M. Takahashi, J. B. Wallingford, M. Kinoshita, Septin-dependent remodeling of cortical microtubule drives cell reshaping during epithelial wound healing. *J. Cell Sci*. **131**, jcs212647 (2018).
